# Progressive Reinvention or Destination Lost? Half a Century of Cardiovascular Tissue Engineering

**DOI:** 10.3389/fcvm.2020.00159

**Published:** 2020-09-09

**Authors:** Peter Zilla, Manfred Deutsch, Deon Bezuidenhout, Neil H. Davies, Tim Pennel

**Affiliations:** ^1^Christiaan Barnard Division for Cardiothoracic Surgery, University of Cape Town, Cape Town, South Africa; ^2^Cardiovascular Research Unit, University of Cape Town, Cape Town, South Africa; ^3^Karl Landsteiner Institute for Cardiovascular Surgical Research, Vienna, Austria

**Keywords:** cardiovascular tissue engineering, history, clinical needs, misleading animal models, refocusing translation

## Abstract

The concept of tissue engineering evolved long before the phrase was forged, driven by the thromboembolic complications associated with the early total artificial heart programs of the 1960s. Yet more than half a century of dedicated research has not fulfilled the promise of successful broad clinical implementation. A historical account outlines reasons for this scientific impasse. For one, there was a disconnect between distinct eras each characterized by different clinical needs and different advocates. Initiated by the pioneers of cardiac surgery attempting to create neointimas on total artificial hearts, tissue engineering became fashionable when vascular surgeons pursued the endothelialisation of vascular grafts in the late 1970s. A decade later, it were cardiac surgeons again who strived to improve the longevity of tissue heart valves, and lastly, cardiologists entered the fray pursuing myocardial regeneration. Each of these disciplines and eras started with immense enthusiasm but were only remotely aware of the preceding efforts. Over the decades, the growing complexity of cellular and molecular biology as well as polymer sciences have led to surgeons gradually being replaced by scientists as the champions of tissue engineering. Together with a widening chasm between clinical purpose, human pathobiology and laboratory-based solutions, clinical implementation increasingly faded away as the singular endpoint of all strategies. Moreover, a loss of insight into the healing of cardiovascular prostheses in humans resulted in the acceptance of misleading animal models compromising the translation from laboratory to clinical reality. This was most evident in vascular graft healing, where the two main impediments to the *in-situ* generation of functional tissue in humans remained unheeded–the trans-anastomotic outgrowth stoppage of endothelium and the build-up of an impenetrable surface thrombus. To overcome this dead-lock, research focus needs to shift from a biologically possible tissue regeneration response to one that is feasible at the intended site and in the intended host environment of patients. Equipped with an impressive toolbox of modern biomaterials and deep insight into cues for facilitated healing, reconnecting to the “user needs” of patients would bring one of the most exciting concepts of cardiovascular medicine closer to clinical reality.

“*Once articles, particularly major reviews, appear that lack historical perspective of discovery, the wheel of reinvention perpetuates itself.”*

Julie Campbell

## Introduction

After years of pioneering work in the 1950 (1–4), the break-through in cardiovascular surgery came a decade later with the ability to replace heart valves (5), repair aneurysms of the aorta (6), and bypass flow-limiting coronary artery stenoses (7).

Continual clinical progress has been made on the basis of better prosthetic materials, better designs and deeper insight into physiological needs. The gradual substitution of polyethylene terephthalate-based (PET) fabrics (“Dacron®”) (8) by expanded fine-fibrillar polytetrafluoroethylene (ePTFE) for medium-diameter grafts improved the patency of arterial prostheses in peripheral bypass surgery (9). Surface structuring (10, 11) seemed to improve the increasingly prohibitive thromboembolic limitations of total artificial hearts (TAH) (12, 13) and glutaraldehyde instead of formalin (14) crosslinking led to a significantly improved durability of bioprosthetic heart valves (15, 16).

By the 1970s, however, the initial belief that cardiovascular prostheses will continually improve began to wane. Pannus formation and thrombo-embolism thwarted the hope that total artificial hearts will make transplantation obsolete (17, 18); synthetic small diameter grafts had distinctly higher occlusion rates than vein grafts (19) and replacement heart valves either prematurely degenerated (20) or caused serious thromboembolic complications (21). It was the realization that no material-based solution can ever match the non-thrombogenicity of the patient's own endothelium that gave rise to what was later called “tissue engineering”. The unifying concept behind this undertaking was to replace diseased parts of the circulatory system with restorative implants which contained or regained the patient's own tissue in order to function like the non-diseased structures they replaced. As this concept integrates biological components with engineering principles and synthetic materials the term “tissue engineering” was coined around a “Keystone Meeting” in Colorado in the 1980s, 20 years after its principles were pioneered for the first time (22–24). For the present review this term will therefore be used for any approach that eventually leads to the creation of living and functional structures of the heart or the vasculature whether through *in-vitro, in-vivo* or combined procedures and as such stretches from single staged cell inoculation to bioreactor based concepts and from the decellularization of cardiovascular organ structures to their induced recellularization.

While tissue engineering soon evolved into an attractive concept for a broad spectrum of indications cardiovascular surgery deserves the distinction of having spearheaded both research and clinical translation for other disciplines.

## Clinical Needs: Then vs. Now

The pioneers of tissue engineering in the 1960s and 1970s were surgeons experiencing the clinical needs first hand. Subsequently the dominance in the field tilted toward basic scientists. Given the fast changing ways cardiovascular surgery has been practiced during the past fifty years, however, this shift away from the surgeons inevitably led to a growing divergence between perceived and actual clinical needs for such implants.

### Original Needs for Tissue Engineering

At the forefront of all tissue engineering efforts stood the pneumatically driven artificial heart. Caused by the dismal results of heart transplants in the pre-ciclosporin era (25), major total artificial heart (TAH) programs emerged globally based on an idea that had been pioneered by Willem Kolff's group in Utah in 1964 (12, 26–29). Apart from Salt Lake City, Houston was a hub for TAH research both at Baylor College and the Texas Heart Institute. Disappointingly, despite attempts to address thromboembolic complications through improved designs (13), the lack of blood-compatibility hampered the success of mechanical blood pumps well into their first long-term use almost 20 years later (30, 31). Therefore, in the absence of a paradigm-changing new immunosuppressive drug for transplantation, creating a living, non-thrombogenic surface lining on these *blood pumps* became a priority in the 1970 and beyond (10, 11, 22–24, 32–36).

In the 1980s, the critical need for prostheses containing functional tissue was in bypass surgery. There, the initial optimism for synthetic vascular grafts had given way to disillusionment. Mid-diameter ePTFE grafts in below-knee reconstructions had a 12% 4-year primary patency in a major prospective multicenter study as opposed to 49% for saphenous vein grafts (37, 38). At the same time, coronary bypass surgery was at its peak as catheter interventions were still an experimental procedure. As arterial grafting was only performed by a handful of surgeons (39, 40), almost all coronary grafts were vein grafts. As a consequence, many of these patients had both their saphenous veins used. Since the 10-year patency of coronary vein grafts is only 45% the reoperation rate in the absence of catheter-based interventions was as high as 14–18% (41). Therefore, with every 6^th^ patients needing a re-operation and often both saphenous veins having been taken due to previous procedures, there was a true, pressing need for synthetic small diameter conduits. Given the dismal results with ePTFE grafts in aorto-coronary position [3-months patency 61% (42)] endothelialised *small-diameter grafts* were the natural focus of tissue engineering efforts during this era.

A decade later, *replacement heart valves* were at a junction. Although repairing the mitral valve was already described in the late 1950s (43–45) and the aortic valve in the early 1960s (46–48) repair-techniques took a back seat for decades to the easily reproducible insertion of a prosthetic device. Disappointingly, the long-term performance of these replacement valves was sub-optimal, either due to complications with anticoagulation in mechanical valves or the fast degeneration of tissue valves in young and middle-aged patients. In the latter group, the 15-year freedom from structural valve degeneration was as low as 31% (49). As such there was a pressing need for better performing replacement valves. The emphasis was on leaflet durability of “soft-leaflet” valves for adult patients who should avoid anticoagulation.

Eventually, at the beginning of the new millennium, progress in bio- and material-sciences allowed to address the longstanding clinical need for *myocardial regeneration*. Although improved medical therapy had almost halved the death rate from heart failure between 1980 and 2000 it was still the leading cause of death in the Western world (only gradually being overtaken by cancer thereafter). Today, with more than 300,000 annual deaths in the USA alone (50)–in their majority due to ischemic heart disease and post-infarction heart failure—myocardial regeneration is the one condition that hasn't lost urgency for clinical tissue engineering solutions.

### Today's Needs for Tissue Engineering

Over the decades, the clinical needs for tissue engineering solutions have changed. Some of them disappeared while others emerged.

In artificial hearts, for instance, positive displacement TAHs with their inverting diaphragms, big inflow cuffs, and prohibitive thromboembolic complication rates have been almost completely replaced by continuous-flow VADS with their equally-dimensioned in- and outflow conduits and active suction replacing passive diastolic filling. Stroke rates in second generation pumps were already down to 11% (51) at a time when Thoratec® the most popular ventricular assist device alone had crossed the 20,000 implant mark. Modern axial devices combined with antiplatelet therapy reached stroke rates of below 5% in spite of manifold longer implantation periods (52).

Even less needed than yesterday's diaphragm-driven total artificial hearts are small diameter vascular grafts for coronary bypass surgery. In today's era at least one–but increasingly more–arterial grafts are being used in each patient. Together with the prevalence of catheter based interventions sufficient autologous grafts are available even in re-operations. Not as extreme but following a similar trend are lower limb revascularizations. By now, endovascular treatments have a nearly universal procedural success rate, low morbidity, and mortality, and with newer devices also improved patency rates. This makes them the recommended therapy of choice, particularly for TASC A–C lesions (Trans-Atlantic Inter-Society Consensus defining the staging of patients with peripheral arterial disease in relation to the expected superiority of either surgical or endovascular techniques). Only in critical limb ischemia (CLI) are surgical vein grafts still superior while prosthetic surgical grafts are associated with even poorer results than contemporary endovascular therapies. Therefore, angioplasty is also recommended as the preferred procedure in patients with CLI who lack an adequate vein conduit (53, 54). As such, the only remaining clinical need for tissue-engineered/regenerating vascular grafts may be for the small number of patients with critical limb ischemia, poor run-off and no saphenous veins available. But here, too, the previously occurring lack of saphenous vein conduits was mostly due to their prior use for coronary bypass grafts, something very unlikely to happen today. It is therefore a dwindling group of patients who would benefit from tissue engineered vascular grafts. Given the fast evolution of endovascular therapies such grafts would need to be based on thin-walled prostheses (55) used in covered stents to avoid that they are obsolete before finding their way into clinical practice. One indication for prosthetic medium-diameter grafts, however, has dramatically grown over the past decades: that for dialysis access grafts. Hardly any other indication saw such an increase in patient numbers from near negligible in 1980 to almost 3 million patients (56) presently being on dialysis globally. Although the Cimino fistula (57) has been the preferred way of creating a relatively longer-lasting access for dialysis puncture, it either prematurely fails or is not possible in a significant proportion of patients. Unfortunately, the alternatively used prosthetic access grafts have a particularly low patency due to thrombosis and neointimal hyperplasia. The primary patency for ePTFE is between 57 and 43% at 1-year and 29% at 2 years (58, 59). Therefore, no other vascular indication would benefit from a superior tissue-engineered graft more than *dialysis access grafts*—provided they not only achieve a surface endothelium but also control intimal hyperplasia.

In heart valves, needs have also changed over the decades. Transcatheter insertion has not only become an acceptable way of replacing diseased aortic valves (TAVI) in high risk patients but is by now the gold standard for the entire spectrum of patients from low- to high-risk (60). As trans-catheter valves are being crimped to small diameters for implantation they depend on crimpable soft-leaflets. Presently, these leaflets are made of bioprosthetic tissue but polymers or other foldable materials that can withstand the mechanical forces during the cardiac cycle are on the horizon (61). As contemporary bioprosthetic leaflets degenerate fast in younger patients (49), TAVIs are currently restricted to patients older than 70 years of age. Therefore, younger patients still receive surgically implanted mechanical heart valves with all their thromboembolic complications (62), even under optimal anticoagulation monitoring. As most of the contemporary TAVIs need the calcium deposits for anchorage they are additionally largely restricted to patients with aortic stenosis. As such, the two unresolved frontlines toward *universal transcatheter heart valve replacements* are stent designs that enable the replacement of both stenotic and regurgitant lesions and crimpable, anti-thrombotic, long-lasting leaflet materials. If both were synergistically resolved, a major clinical need for all non-repairable symptomatic patients younger than 70 years of age would be addressed. As a second field for tissue engineered heart valves, needs have grown in *congenital cardiac surgery*. The population of adults with congenital heart disease (CHD) has grown steadily. Having reached an estimated 1.3 million in the United States, adults with CHD are now more numerous than children and constitute 60% of the total CHD population (63). As much as growth is always emphasized as a main motivation for “living” heart valves in children, it is in fact also the longevity of the leaflets that dominates the needs. Growth does not play a major role on the left side of the heart as it is largely the annuli which are the size-restricting structure. For the aortic valve, this problem is addressed by the Ross procedure where a patient's own pulmonary valve is used as an autograft for replacing a diseased aortic valve while using an allograft for the biomechanically less strained pulmonary valve. Indications on the right side are those currently requiring homografts–from Ross procedures to Tetralogy of Fallot with pulmonary atresia; from truncus arteriosus to double-outlet right ventricle. Here, tissue engineered solutions would meet needs both at the original operation to avoid homograft failure after years and at the time previously implanted homografts fail and present for re-intervention. The latter would again be better served by a tissue-engineered trans-catheter solution as stenotic failed homografts typically lead to enlarged right ventricles that could add morbidity and mortality to the re-entry at open heart surgery. There is additionally a pediatric need for large tissue engineered ***patches*** for diameter augmentation of the aorta or the pulmonary artery/ies.

Although annual needs for replacement valves by far exceed a million patients (64, 65) and >3 million patients are in need of dialysis access, the highest number of patients potentially benefitting form a tissue engineering approach would still be those with congestive chronic heart failure (CHF). Over the decades, the number of patients with chronic heart failure has exponentially increased due to prolonged life expectancies and the progressive nature of cardiovascular diseases (66, 67). Today, more than 5 million patients are affected in the USA alone (68, 69). Their mortality is >25% within the first year of diagnosis (70, 71). Chronic heart failure is a very serious disorder in children too, and one- third of the children die or receive a heart transplant in the first year after diagnosis (72). Heart transplantation as the ultimate treatment option for this growing group of patients cannot meet the needs leaving the *replenishment of lost cardiomyocytes* with the goal of structural and functional heart muscle repair as the only alternative to mechanical support.

Overall, while total artificial hearts and small diameter vascular grafts were the holy grail of tissue engineering at its outset today's needs are mid-diameter vascular grafts for dialysis access shunts, heart valves, and myocardial regeneration. Given the fact that first attempts to create functional, living tissue as part of a cardiovascular prostheses occurred more than 52 years ago, developments may again outpace progress resulting in the redundancy of a solution when it eventually fulfills clinical expectations.

## A History of Reiterations

Nothing epitomizes the slow progress of cardiovascular tissue engineering more than its reiterations over the course of half a century.

In order to generate functional, non-thrombogenic autologous tissue on cardiovascular prostheses, autologous cell inoculation was first reported by de Bakey's group in 1968 (73), the use of cell-culture techniques by Mansfield (74) in 1968 and Bernhard et al. (75) in 1969 and the induction of fall-out healing from circulating cells by Ghidoni in 1968 (76). The principles pioneered in these early days kept recurring over half a century. The reasons for the near *de-novo* rediscovery of similar basic approaches in 10 to 20 year cycles were manifold. One was certainly the fact that different interest groups in cardiovascular surgery discovered the potential of tissue engineering during different eras. Originally driven by artificial heart programs to address the frustration with a seemingly insurmountable thromboembolic complication rate in the 1960s and 1970s, vascular surgeons took over the baton in the 1980s and 1990s to improve the patency of small diameter grafts followed by cardiac surgeons resuming the lead again with a focus on prosthetic heart valves from the 2000s onwards overlappingly with the efforts of cardiologists and later engineers and material scientists to induce myocardial regeneration in the 2000s and 2010s. Each of these eras started with immense enthusiasm but was at best only remotely aware of the preceding efforts. Another reason for the cyclic rediscovery of tissue engineering for different cardiovascular prostheses was the lack of break-throughs leading to a true change of clinical practice. Had the early efforts led to a stable non-thombogenic endothelial lining of artificial hearts, both small diameter vascular grafts and prosthetic heart valves would have been pursued in continuity building on the previous developments. Moreover, as Julie Campbell put it (77), once articles, particularly major reviews, appear that lack historical perspective of discovery, the wheel of reinvention perpetuates itself.

## Successes and Failures

Over the decades, principles have not much changed but re-emerged under different names. What did, however, genuinely change was our understanding and interpretation of the biological processes involved. Circulating progenitor cells, homing mechanisms and trans-differentiation have gained traction over phenotypical determination, mass-harvest and mass-culture. The perhaps biggest impact had the recognition of trans-differentiation, a pathway that was still highly contested in the 1970s (78). The concept was an affront to the eminent paradigm of lineage-based developmental biology by which cells reach a terminally differentiated state. Its impact was felt in early vascular tissue engineering. The “purity” of cultured endothelial cells (79) for instance, was seen as key to the clinical success of vascular graft endothelialisation as “contaminating” smooth muscle cells were assumed to be the nucleus of occlusive intimal hyperplasia. It took long-term clinical explants to disprove this paradigm. The clinically implanted “pure” endothelial monolayers had in fact developed into a neo-artery structure with mature, contractile smooth muscle cells underneath the endothelium, separated by a well-developed internal elastic membrane (80). As trans-anastomotic outgrowth and trans-mural ingrowth could be excluded in these grafts the two likely retrospective explanations are either homing of circulating cells or trans-differentiation of the originally implanted cells. Since then, the immensely promising field of stem cell and progenitor cell (trans)-differentiation opened the door to a new era with post-lineage trans-differentiation being equally exciting. There, trans-differentiation from adipocytes into epithelial cells (81), fibroblasts into endothelial cells (82) and vice versa endothelial cells into fibroblasts (83, 84) has been demonstrated. Yet, macrophages hold center-stage in this field, having been shown to transition into fibroblasts (85), myofiboblasts (86, 87), and vascular smooth muscle cells (88–91). The boundary between stem cell / progenitor cell differentiation and post lineage trans-differentiation is still blurred as the role of circulating monocytes as endothelial progenitor cells underscores (92). The acknowledgment of trans-differentiation went hand in hand with that of the role of biomechanics (93). Its recognition as a main regulator of intimal hyperplasia in vein grafts on the tissue level (94–98) was as important as that on a cellular level where mechano-transduction for instance proved to be key to the differentiation of stem-cells and progenitor cells (99–101). Vein graft intimal hyperplasia highlighted the potentially detrimental effect on the clinical performance of tissue engineered implants if the biomechanics–including shear forces–deviate from the needs of the destination site (94–98, 102). Similarly, changes in substrate stiffness had been shown to lead to stem cells differentiating toward different lineages (103) providing one of many potential explanations for e.g., tissue engineered heart valves ending up with fibrosing and shrinking leaflets instead of delicate tri-layered replicas of nature (104, 105) or insufficient remodeling of vascular grafts (106).

As none of the tissue engineering approaches of the past 52 years led to a change in clinical practice they reflect half a century of disappointment and only partially fulfilled promises. While each individual concept had specific issues (lack of continuity; too low a cell inoculum; too slow or too fast scaffold degradations; biomechanical incompatibilities or remnant immunogenicity) one single root-failure in the clinical translation can be identified as a common thread: the refusal to accept or recognize that the trans-anastomotic healing mode that leads to successful *in-situ* endothelialisation in animal models is wholly irrelevant in man.

## Collective Complicity

As much as modern laboratory science allows to answer many questions *in-vitro*, the peculiar healing characteristics of cardiovascular implants in man make animal implants unavoidable. While animal models cannot fully emulate the human situation in general, the ones used for cardiovascular implants have been particularly unsuited to provide accurate pre-clinical feed-back. As such, they were and still are the main obstacle to the clinical translation of cardiovascular tissue engineering. In animal research, it does make a difference whether a well-considered model for clearly defined questions can still only partially provide appropriate answers or if a broad–though unwitting–consensus prevails to use established animal models out of convenience, skills- and cost reasons regardless of the fact that the gap to the clinical reality is unbridgeable.

**Figure 1:**
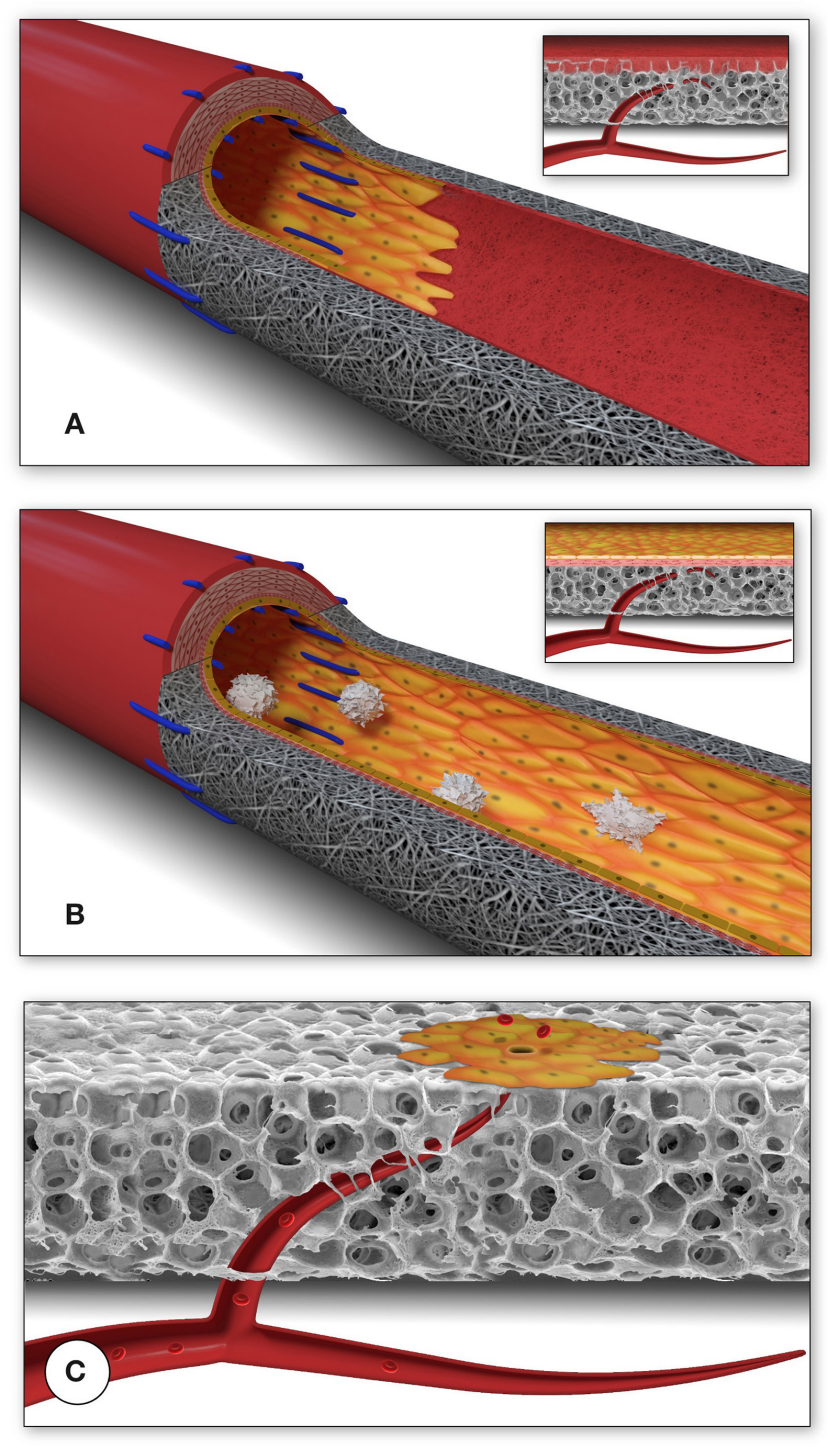
Schematic presentation of the fundamental difference in cellularization and healing of prosthetic cardiovascular implants in humans **(A)** and in animal models **(B)**. In humans, transanastomotic outgrowth hardly exceeds a few millimeters even after years of implantation. Continual fibrinogen and platelet replenishment from the blood leads to a compacted surface thrombus in the luminal interstices of the scaffold that increasingly becomes hostile toward capillary penetration similar to the wall thrombus of aneurysms. Over time, this compacted acellular material in the luminal layers of a scaffold becomes prohibitive for transmural endothelialisation (Insert A) even if scaffold structure and/or degradability would facilitate capillary penetration. The rapid trans-anastomotic outgrowth of adjacent endothelium and its subintimal cells in the vast majority of animal models **(B)** also mitigates transmural vascularization (Insert B) while actively recruiting cells from the circulation. As such, the entire healing pattern in most animal models from surface endothelium to intramural cell population is non-predictive for the tissue response in patients. For transmural endothelialisation to be successful **(C)**, models need to be chosen where the presence of a surface endothelium is not pre-empted. Only this allows to study the antagonistic dynamics between ingrowth spaces, accelerated angiogenesis and the build-up of increasingly impenetrable, compacted thrombus in the luminal interstices of a scaffold.

**Figure 2:**
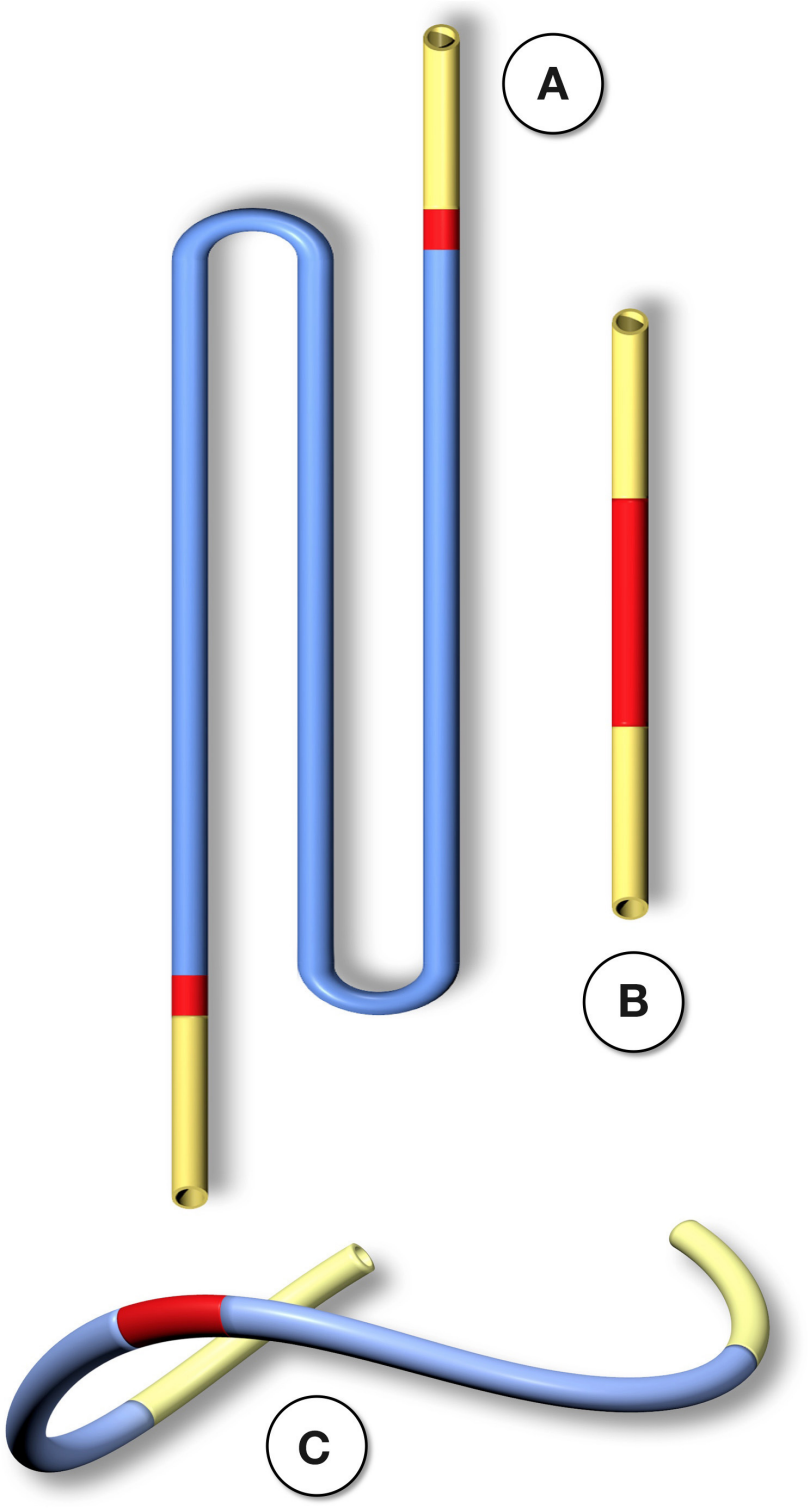
Schematic comparison of graft lengths typically used for clinical peripheral bypass grafts **(A)** and experimental grafts implanted in animals **(B)**. In >90% of all large animal experiments grafts were shorter than 6 cm. In most animal models, trans-anastomotic endothelial outgrowth (red) leads to complete surface endothelialisation within weeks making it impossible to study transmural or blood-born endothelialization. In clinically implanted bypass grafts, in contrast, ingrowth stoppage permanently leaves over 90% of the graft non-endothelialized (blue). Therefore, animal studies generally investigate a mode of surface endothelialization that is irrelevant in humans. To study transmural- or fall-out endothelialisation from the blood stream experimental grafts need to be welded between sufficiently long low-porosity grafts to clearly see a non-endothelialized zone between the progressing margins of transanastomotic outgrowth and endothelium originating from the investigated mid segment **(C)**. From (102) with permission.

In humans, trans-anastomotic endothelial outgrowth plays no role as it does not exceed more than a few millimeters even after years of implantation (102, 107–109) (Figures 1, 2). As such, it is evident that in patients, any *in-situ* tissue engineering approach would need to harness either transmural endothelialisation or fall-out healing. Yet, by choosing short graft lengths in rapidly endothelializing animal models, implants were often trans-anastomotically fully endothelialised by the time of explantation (102, 110) (Figures 1, 2). This was aggravated by the fact that in over 90% of large animal studies the average graft length was <5.5 cm (102). Taken into consideration that ingrowth occurs from two anastomotic sites the de facto graft length was 2.8 cm in animal models where the trans-anastomotic outgrowth is a few centimeters per month! To put it into perspective: the same outgrowth distance which takes 56 weeks in humans before it comes to a complete stop is reached after 3.5 weeks in dogs and significantly earlier in other species (102). Although it is understandable that abandoning well-established models would have been highly disruptive from the standpoint of implantation skills and laboratory-specific data histories, this system-immanent hurdle to the clinical translation of tissue engineering is also potentially dangerous when it comes to misleading preclinical results that may prompt premature clinical trials.

## Limits of Engineered Tissue Regeneration in Humans

The *in-vivo* population of scaffolds with host tissue is a critical component of tissue engineering whether or not cells were already incorporated at the time of implantation. Naturally, cell-free implants rely entirely on the *in-vivo* in-growth or outgrowth of host tissue. Given the crucial role of this process it is puzzling how underappreciated the question remained whether the presumed mode of “healing” can be expected to successfully occur in humans.

After half a century of a de-facto focus on the largely irrelevant surface outgrowth of tissue across the anastomoses, experimental data will need to have answered three questions before they can be assumed to succeed in their clinical translation: (a) was an experimentally demonstrated tissue regeneration mode likely to happen at the intended clinical site; (b) is the successful population of an implant with all functional tissue components likely to happen in humans before the build-up of adverse ingrowth conditions and (c) in the case of degradable scaffolds–can functional tissue formation be completed before the scaffold starts disintegrating.

**Figure 3:**
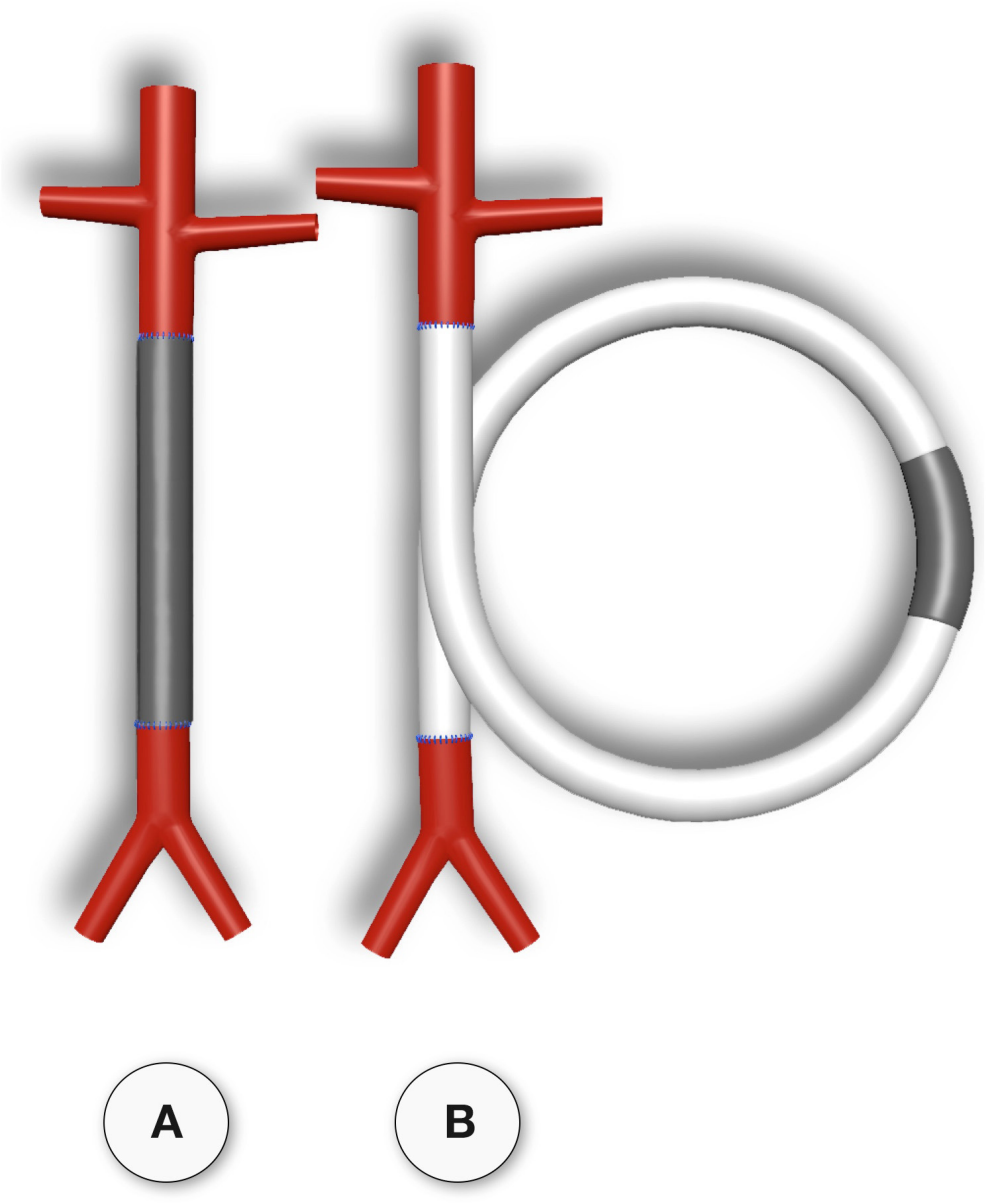
Typical straight infra-renal interposition graft in rats usually ± 10 mm in length **(A)** and loop graft of ± 100 mm length with an experimental segment welded into the mid-region of low-porosity ePTFE **(B)**. While straight infrarenal interpositions usually lead to.trans-anastomotic endothelialisation before trans-mural ingrowth can occur the “isolation” segments in the loop graft enable the investigation of transmural endothelialisation without interference from transanastomotic endothelialisation. By also sealing the interposition segment against the adventitia, a model for fall-out healing is created. From (110) with permission.

Although experimental models that excluded trans-anastomotic endothelialisation were proposed >35 years ago when Hess et al. (111) suggested a looped conduit that enables the implantation of graft lengths of up to 10 cm in the infrarenal aorta of the rat they remained in obscurity until recently (110, 112, 113) (Figures 2, 3).

Of all modes of cell population, *facilitated fall out healing* from the circulation would be the holy grail of tissue regeneration. It is therefore surprising that not more efforts had gone into clarifying the true potential of this healing mode. Again first described by DeBakey's group in Houston in 1963 (109, 114) and clinically confirmed by Berger and Sauvage at the University of Washington in the early 1970s (107) “fall out” endothelialisation had been sporadically observed thereafter both on artificial hearts (35) and on vascular grafts (113, 115–121). Key to being able to observe true “fall-out” healing without interference from either trans-anastomotic or trans-mural ingrowth, however, are double isolation models that clearly distinguish it from all other possible regeneration modes both from the adventitia and the anastomotic side. It mystifies that isolation models capable of achieving this periodically emerged over the past four decades (87, 111, 113, 117) without enthusing the countless groups that would have needed this healing mode for the validation of their tissue engineering concepts. Almost 40 years ago—also in the context of artificial heart research–Feigl et al. already used such a double-isolation model to unambiguously prove in large animals that facilitated surface entrapment of circulating mononuclear cells can lead to their differentiation into a neo-vessel structure containing myofibroblasts and endothelial cells within a PET scaffold (87). Most recently, the occurrence of fall-out healing was again confirmed in a murine loop-graft isolation- and sealing-model (Figure 3) which did reemphasize how minor the contribution of this healing mode is to surface endothelialisation as opposed to transmural endothelialisation (113).

**Figure 4:**
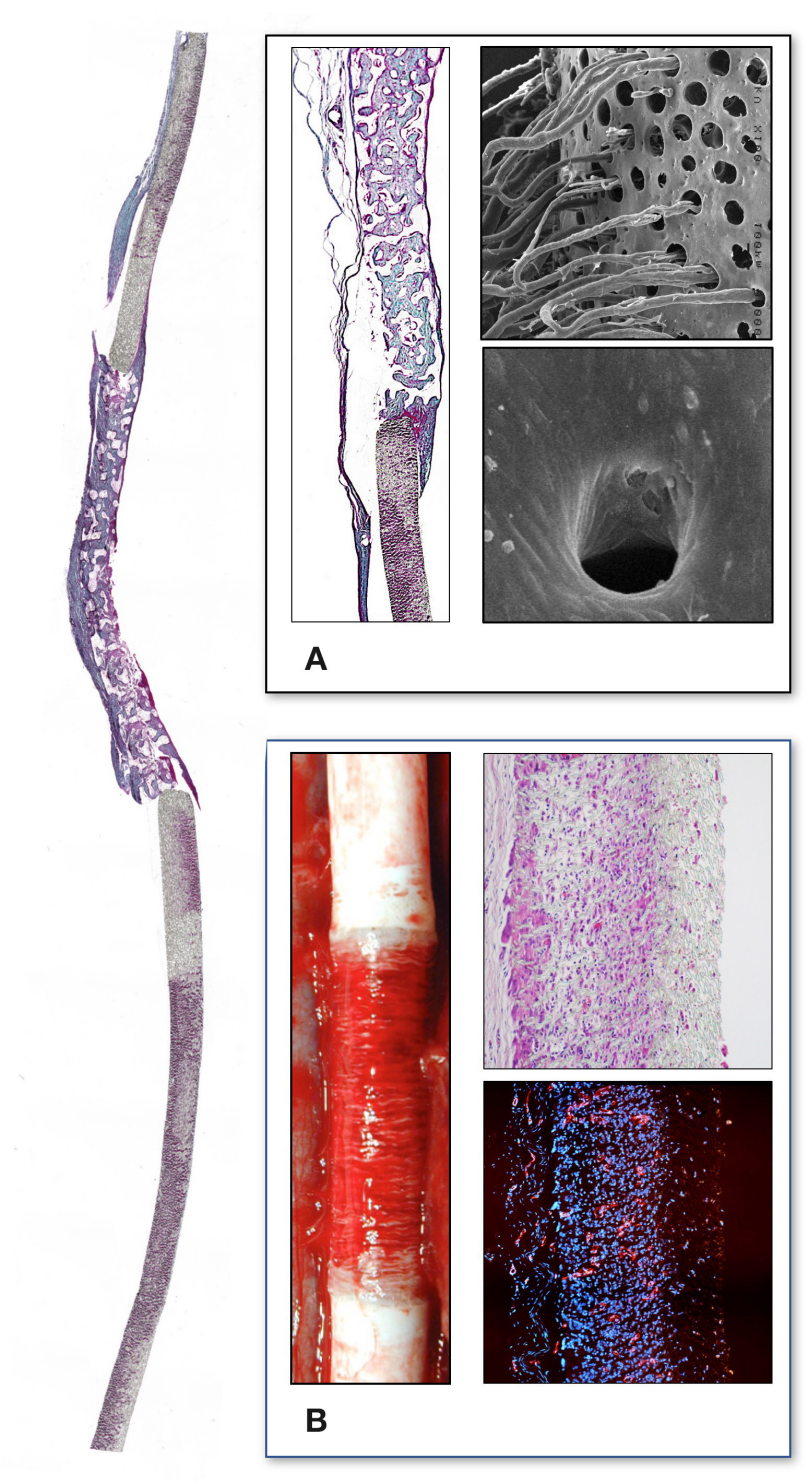
Interposition isolation models for studying endothelialisation without the interference of trans-anastomotic endothelial out- and over-growth. **(A)** Rat infra-renal loop graft model. A high porosity polyurethane graft was welded into the mid-segment of an up to 100 mm long low-density ePTFE graft. The confluent mid-graft endothelium reached onto the otherwise endothelial-free ePTFE sections. Transmural ingrowth from the adventitial side was confirmed with corrosion casting. The origin of the surface endothelium was sometimes traceable to capillary openings on the blood surface. **(B)** Senescent baboon femoro-femoral isolation graft model. Experimental grafts were equally welded into the mid-section of low-porosity ePTFE. The experimental ePTFE graft shown possessed a dense middle layer which made it impenetrable for cells. After 6 weeks, cellularity was almost exclusively on the adventitial side, highlighting that trans-mural ingrowth from the adventitia overwhelmingly accounts for the cell population of the graft wall unless actively recruited by transanastomotic endothelium (see Figure 6). From (113) and (102) with permission.

**Figure 5:**
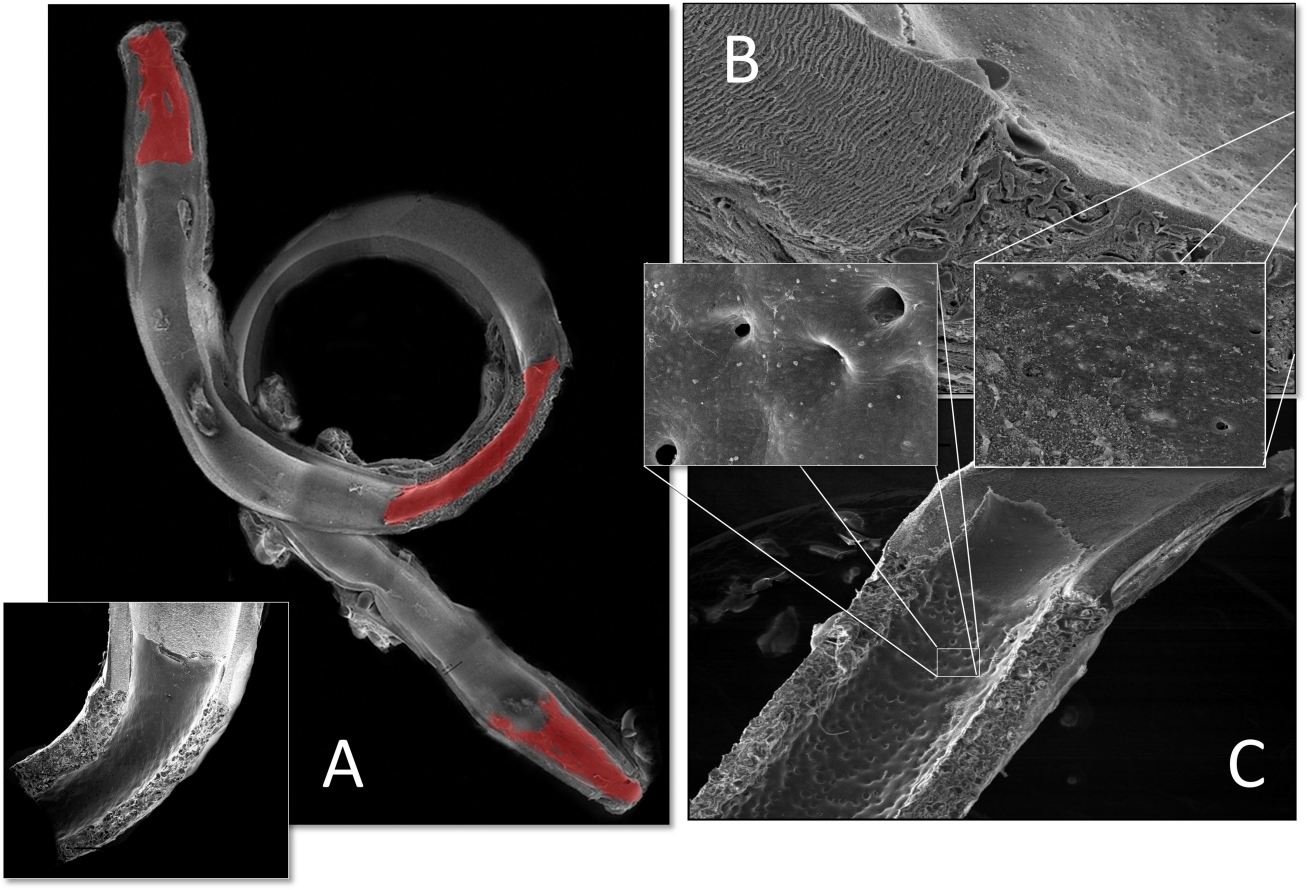
Transmural endothelialisation of vascular grafts in the rat **(A)** and the senescent baboon **(B,C)**. The loop graft in the rat **(A)** shows clearly discernible edges of the trans-anastomotically outgrowing endothelium (post-colored in red) from the infra-renal aorta with a long stretch of endothelium-free surface separating it from the trans-mural mid-graft endothelium (also post-colored in red). The presence of a long separating zone between the experimental graft and the transanastomotic outgrowth-edge is even more important in view of transanastomotic outgrowth also occurring in the opposite direction (Insert A) from the experimental graft in case of successful trans-mural endothelialisation. **(B,C)** Similar isolation graft in femoro-femoral position in the senescent Chacma baboon. After 6 weeks, transmural sprouting had either successfully led to confluent surface endothelialisation **(C)** with multiple capillary openings (Insert C) or in its absence led to the build-up of a dense fibrin matrix in the interstices near and on the surface **(B)**. Sometimes sporadic small endothelial islets are detectable (Insert B).

Thus, in the absence of any foreseeable breakthrough with facilitated “fall out” healing in the near future, *transmural endothelialisation* will remain the only likely avenue to a successful clinical *in situ* endothelialisation particularly of synthetic cardiovascular prosthesis. Key to this healing mode first described in 1962 (122) is the penetration of the full wall thickness of an implant by sprouting adventitial capillaries. These tubes were shown to coalesce with the graft lumen on the blood surface (113, 123–125) where they give rise to expanding endothelial islands (102, 113) (Figures 4, 5). Not only do these transmural vessels largely maintain continuity with the abluminal side (123, 127), they are also likely to attract other cell types required for the complete tissue integration of the scaffolds. As Patricia d'Amore had already concluded in the 1990s undifferentiated mesenchymal cells may follow these endothelial tubes and be directed by them in their differentiation into SMCs (128). Although facilitating circumstances such as endothelial-rich peri-graft tissues were shown to potentially augment transmural endothelialisation (129–131) the scaffold itself must allow this process by providing continual, ingrowth-permissible spaces (102).

**Figure 6:**
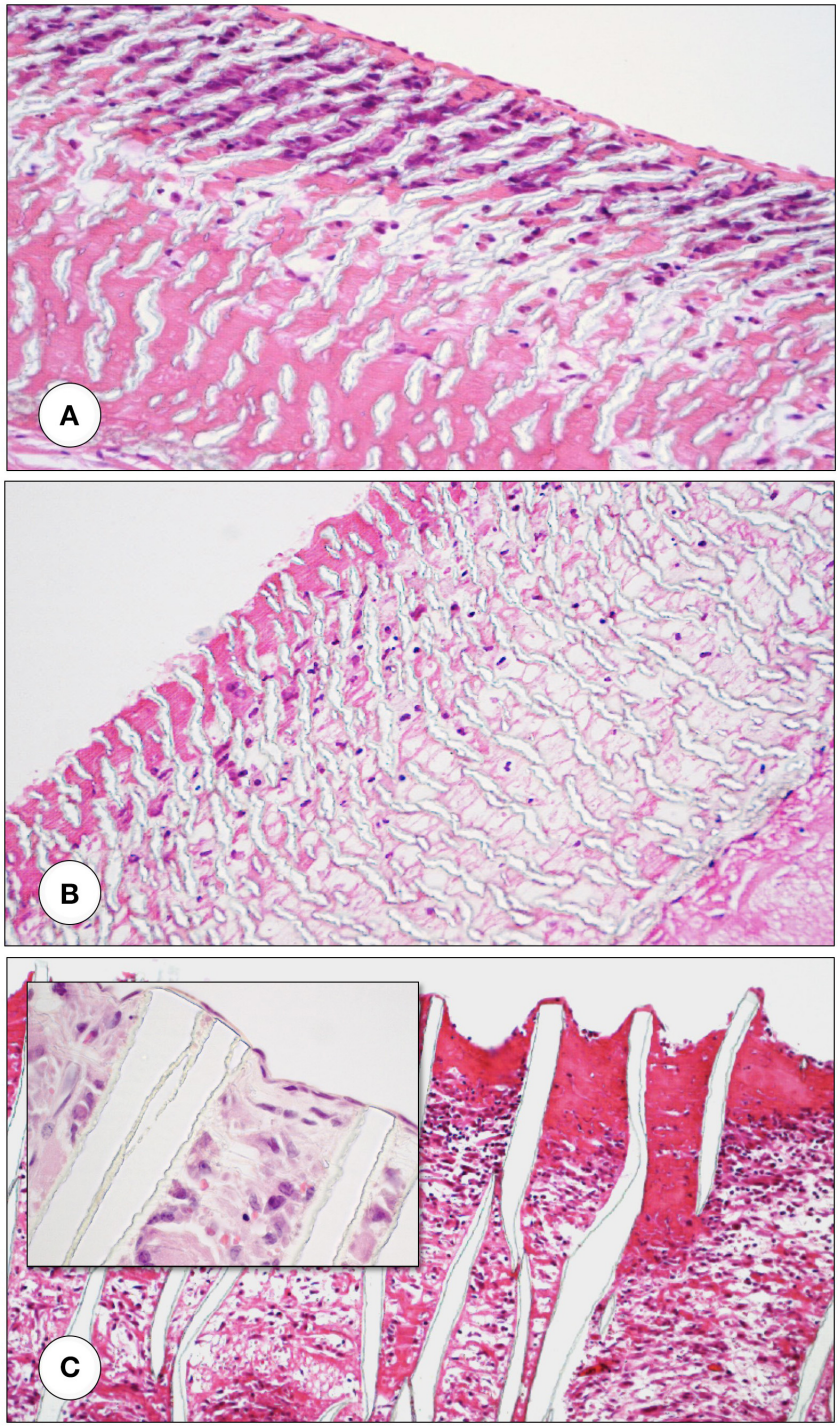
Femoro-femoral isolation model in the Chacma Baboon (6 weeks) (102, 126). The wrapped, ingrowth-preventive 30 μm ePTFE segements [**(A)** near anastomosis with trans-anastomotic endothelial outgrowth; **(B)** beyond trans-anastomotic endothelium with compacted surface thrombus)] had a high-porosity 150μm IND experimental ePTFE graft [**(C)** and Insert] welded into the mid-section. The low-porosity, wrapped isolation segments highlight the need for the presence of an endothelium for the active recruitment of largely mononuclear cells from the blood stream **(A,B)**. The very-high porosity isolated mid-graft segment showed either dense, compacted thrombus in the internodal spaces of the luminal side **(C)** or well-healed grafts with fully trans-murally endothelialized blood surfaces (insert C).

**Figure 7:**
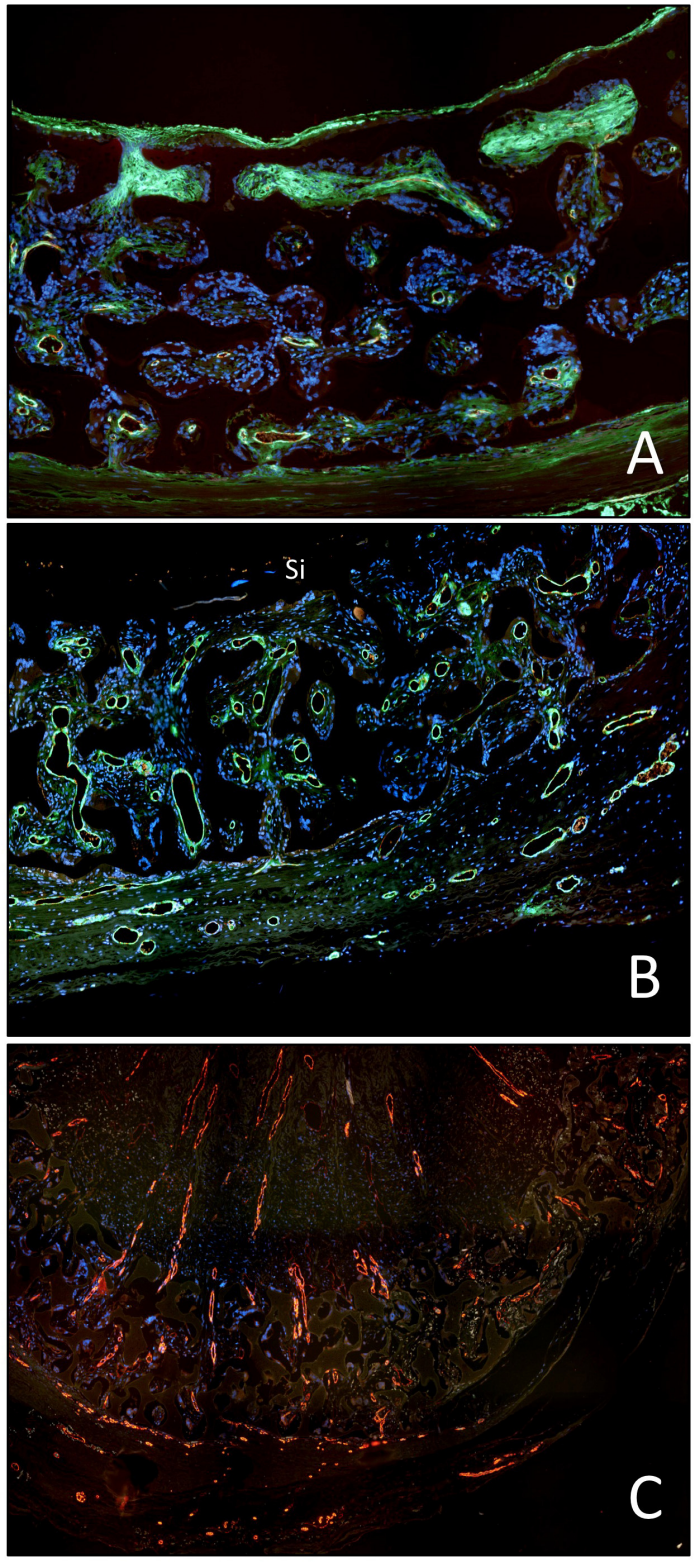
Demonstration of the detrimental effect of the compacted, dense surface thrombus on transmural endothelialisation in the baboon femoro-femoral isolation model (identical implant periods in A to C). **(A)** Restriction of transmural vessel ingrowth to the outer half of the graft in the presence of a compacted surface thrombus in the interstitial spaces of the luminal third of the graft wall. **(B)** Identical mid-graft with a sealing, oxygen-permissible silicon membrane on the blood surface. The transmural vessel ingrowth reaches through the entire wall thickness. **(C)** Identical graft as in A but occluded. The long-distance outgrowth of trans-mural blood vessels through the graft wall and the entire thrombus highlights the fact that fibrin clots are pro-angiogenic unless they become compacted near the blood surface as typically seen in non-endothelialised vascular grafts in humans.

Under clinical circumstances, however, transmural endothelialisation is not only determined by ingrowth spaces but also by two interdependent *in-situ* responses distinctive to humans: the near absence of trans-anastomotic endothelialisation (Figure 2) and the resulting gradual build-up of impenetrable thrombus near the blood surface over time (Figures 5–7). While trans-anastomotic outgrowth-inhibition has been a well-known phenomenon for the past 60 years (107) the detrimental effect of the resulting surface thrombus-compaction on transmural tissue ingrowth has only slowly emerged. Observed from the 1960s onwards (102, 107–109, 115, 116, 132, 133) this acellular dense fibrin layer building up in the interstices of synthetic vascular grafts near their non-endothelialised blood surfaces was initially thought to be PET related (109, 134, 135). Yet, resembling the almost acellular “anion-layer” thrombi of aneurysms (136) comparative primate studies in the 1980s already suspected the platelet-rich, dense fibrin itself to be the culprit (135). Subsequent insight into the diversity of thrombus formation eventually provided some explanations in the 1990s (137). While a typical “wound healing type” fibrin with its relatively low fiber density and thick fibers strongly stimulates angiogenesis (Figure 7C) the high-density mat of thin fibers precipitating in a fibrinogen rich environment like the one near the surface of cardiovascular implants is more thrombogenic (133) and inhibits capillary formation (138) (Figures 5B, 6C, 7A,B). As such, complete transmural healing needs to be concluded before this barrier builds up (102). As was shown in an isolation model in a non-human primate (102, 126) non-facilitating circumstances may allow some individuals to successfully conclude transmural endothelialisation before the build-up of hostile surface thrombus while in others sprouting capillaries already hit a barrier (Figure 6C). The focus of engineered tissue regeneration efforts must therefore be to temporarily block the build-up of impenetrable interstitial thrombus near the blood surface while facilitating accelerated transmural endothelialisation.

## A Protracted Evolution

Comprising the experience of more than five decades, concrete tissue engineering approaches ranged from direct cell inoculation to bioreactor-based *in-vitro* culture; from decellularization of natural tissues to purely synthetic concepts; from degradable fabrics to injectable scaffolds and from facilitated ingrowth to inadvertent outgrowth. As these sometimes novel and at other times reiterated strategies emerged over more than half a century, a systemic classification never materialized. Yet, the clearest overarching order seems to be one that distinguishes between “vital” and “non-vital” implants.

### Vital Implants

#### Single-Stage, Direct Inoculation of Autologous Cells, or Tissue

Naturally, the inoculation of prostheses with autologous cells at the time of surgery represented the first step toward “living” implants.

Given the insurmountable thromboembolic complications of artificial hearts and the conclusion of deBakey et al. (109) and Wesolowski et al. (108) in 1964 that prosthetic blood surfaces remain non-endothelialised in humans it was foreseeable that first efforts toward tissue incorporation would be made at leading centers for artificial heart research. Accordingly, it was Ghidoni of deBakey's group who inoculated fragments of autologous skeletal muscle into the velour lining of left ventricular bypass pumps leading to foci of proliferating spindle-shaped cells but no endothelium after 2 weeks (139)—the longest possible implantation period before these early pumps failed. The attached tissue stripes sowed central necrosis and the concept was abandoned in favor of using cultured cells.

Fifteen years later, Mid-Western vascular surgeons from Indianapolis (140) and Ann Arbor (141) took up the idea of autologous single-stage cell inoculation and applied it to Dacron prostheses calling it “Endothelial Cell Seeding.” In dogs, seeding of venous endothelial cells successfully generated a mid-graft endothelium (141). Yet, when carried over into clinical trials, venous endothelial cell-seeding failed to achieve any improvement to the patency of the up to 60 cm long, medium-diameter ePTFE prostheses used for femoro-popliteal bypass grafts (142). It became obvious that seeding densities were too low, aggravated by a reduced reproductive capacity of endothelial cells in smokers (143–145) and patients with hyperlipidaemia (146, 147)- two conditions present in the vast majority of patients. Attempts to increase harvest efficiencies by using microvascular endothelial cells from adipose tissue (148, 149) or bone marrow (150, 151) again led to the successful creation of a functional endothelium in animal experiments (151–153). Yet, microvascular seeding raised concerns regarding unrestricted subendothelial proliferation (154) and when it was eventually clinically tested it also showed no significant improvement over controls (155). Typical for tissue engineering in general, single-staged seeding was rediscovered 25 years after it was first described (156, 157) and by a few researchers is still carried over into the present time (158–161). The main novelty was the replacement of the bio-stable PET and PTFE scaffolds by bio-degradable materials (156). Using again bone marrow as a cell source the concept was experimentally tested in vascular grafts in dogs (151), sheep (162), pigs (163), and mice (160) followed by a clinical pilot study in children (157). When seeded tube grafts were implanted as extracardiac total cavo-pulmonary connections (TCPC) significant graft stenoses developed in more than a quarter of all patients (159, 164). Experimental work suggested that higher seeding densities may mitigate graft stenosis (160, 161). Alternatively, pharmacological interventions were tested emulating the previously tried ACE inhibitor therapy (165) with modern angiotensin II receptor blockade (166). Eventually, single stage seeding of degradable scaffolds was carried over to heart valves and tested in acute (167) and semi-acute (168) sheep implants. Like 37 years before (140), cells were embedded in a fibrin matrix deep within the polymeric meshwork at the time of implantation. When bone marrow cells were seeded on supramolecular polymers, long term results were discouraging with valves failing due to tissue overgrowth and leaflet fusion (158). When the same scaffolds were used as unseeded valves, results were significantly better (158).

**Figure 8:**
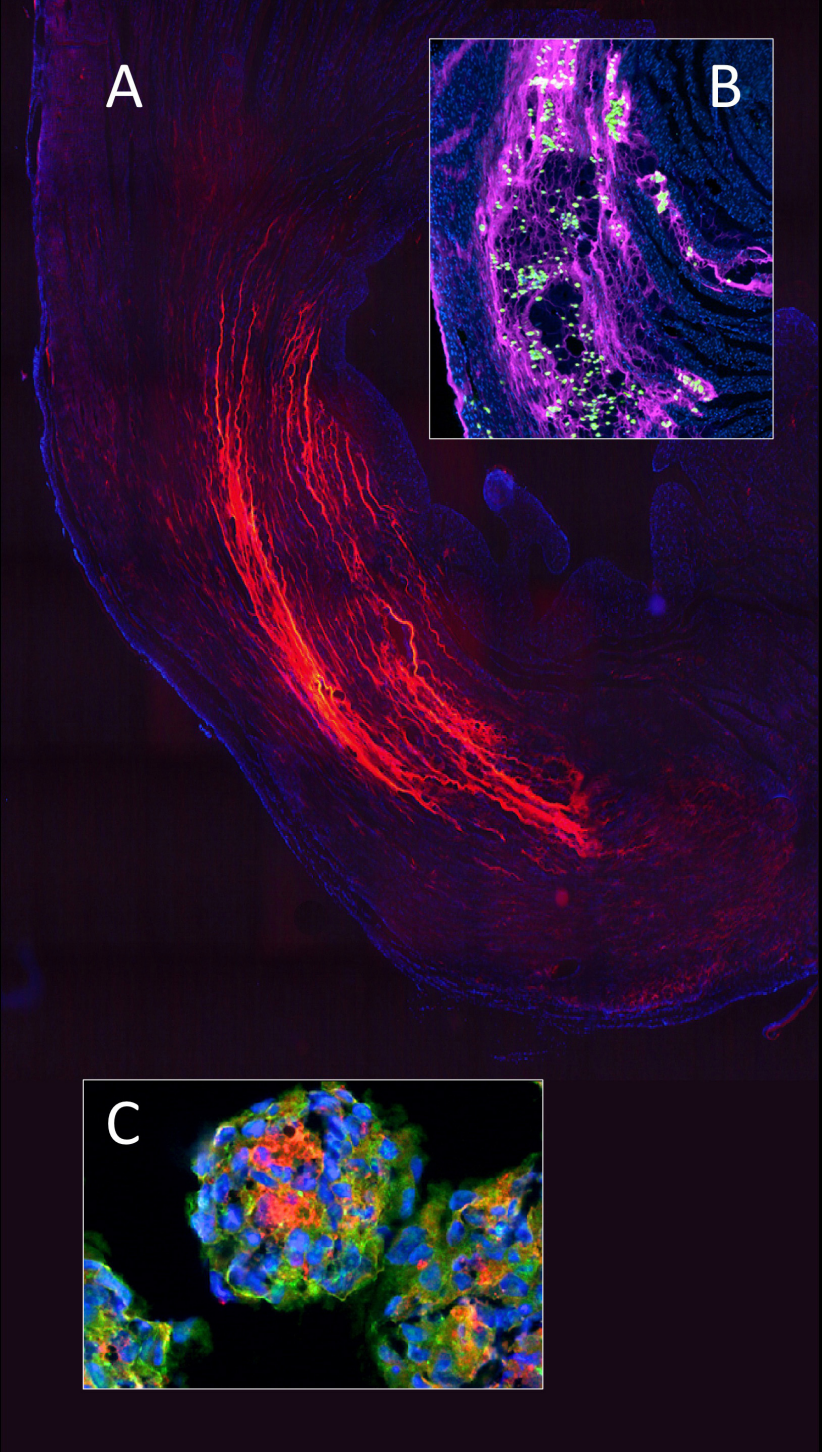
In myocardial regeneration, the potential of a hydrogel (polyethylene glycol) to preserve space and entrap cells is demonstrated with fluorescent labeled PEG (Alexa 665 nm) seen polymerised between the cardiomyocyte bundles in an infarcted rat heart **(A)**. The advantageous effect on stress reduction through wall remodeling was much more pronounced if the gel injection was delayed. Inset **(B)** shows a similarly labeled PEG hydrogel entrapping adipose derived mesenchymal stem cells (green) within the infarcted wall of a rat heart. To improve cellular retention in myocardial regeneration therapy, cellular self-assembly into 3D microtissues (3D-MTs) using the “hanging drop” method (178, 179) prior to intra-myocardial injection **(C)** and compact 100 μm thick myocardial muscle bundles grown from human induces pluripotent stem cells (hiPSC) (176) or human embryonic stem cells (hESC) (177) have emerged as an encouraging alternative to single cell injection “therapy” with its high cell loss due to a lack of entrapment. 3D-microtissues have been shown to significantly enhance the angiogenic activity and neovascularization potential of stem cells. From (180) and (179) with permission.

**Figure 9:**
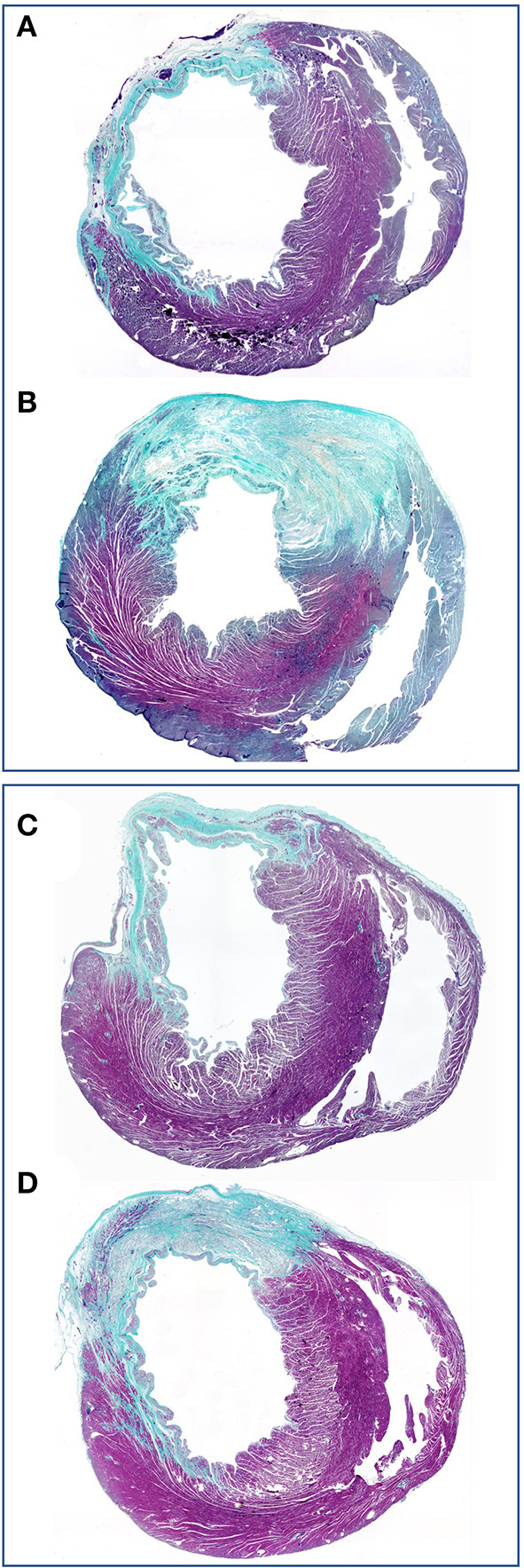
Concomitant stress reduction through gel injection in post infarction myocardial regeneration. The preservation of wall thickness in infarcted rat hearts after injection of polyethylene glycol hydrogels is clearly visible at 4 weeks **(B)** and 13 weeks **(D)** relative to untreated controls **(A,C)**. Finite element models (185, 186) have shown that this mitigation of detrimental post-infarction remodeling dramatically reduces the ventricular mechanical stress (187) that drives the infarcted heart toward failure. Green (scarring) and purple (viable myocardium). From (183) with permission.

Eventually, the ultimate exploit of single-staged cell inoculation was in myocardial regeneration where it endured the longest. This was partly because the clinical drivers were not surgeons and as such least likely to have been exposed to the disappointing previous efforts. At the same time, the enthusiasm for single-staged myocardial cell inoculation was certainly also carried by its concurrence with the early days of stem cell research and the accompanying fascination with the potential of these cells. Early experimental reports describing startling myocardial regeneration in mice after MI (169) triggered a global stampede toward myocardial regeneration work resulting in clinical trials being reported within a year (170, 171) just to be thoroughly disproven 2 years later (172). Fifteen years after the first gung-ho period, meta-analyses of clinical trials with adult stem cells still showed conflicting results ranging from minimal to no therapeutic benefits (173, 174). The greatest shortcoming of the initial approach was the naïve misconception of myocardium as a gel-like structure rather than the dense sponge which particularly the venous side of the vasculature constitutes. As such, rather than engrafted, the injectates were largely washed out and the few remaining cells did not find a matrix environment to survive (175). Recent approaches toward defeating this Achilles heel have been the alternative use of compact 100 μm thick myocardial muscle bundles grown from human induced pluripotent stem cells (hiPSC) (176) or human embryonic stem cells (hESC) (177) or the injection of large cell agglomerates in form of “micro tissue” spheres (178, 179) (Figure 8) and the use of injectable biomaterial scaffolds to entrap and nurture the cells after delivery which has started to achieve incremental improvements (181, 182). Apart from embedding transplanted cells in a matrix that contains engraftment and pro-angiogenic cues, the second benefit of injecting such gels into myocardial infarcts is as a space-holder arresting an otherwise deleterious downward spiral of remodeling (183, 184) (Figure 9). Moreover, the initial hope to facilitate the trans-differentiation of progenitor cells to cardiomyocytes has given way to a more modest expectation allowing for anti-fibrotic and anti-inflammatory outcomes on the basis of a paracrine effect regardless of the cell types inoculated (188). In a translation context, exploiting the components of a paracrine mechanism, particularly exosomes is attractive due to reduced regulatory requirements and their off-the-shelf nature (189). Notwithstanding, the efficacy of this approach will again rest on efficient delivery perhaps most likely through controlled release from injectable scaffolds or sophisticated targeting approaches (190).

In hindsight, the trajectory of myocardial regeneration, however, hasn't been different from other areas of cardiovascular tissue engineering. Although much basic knowledge had been gained in the three preceding decades, this latest embodiment of the earliest approach to tissue engineering repeated many of the mistakes and equally fell for the temptations of its predecessors: hype at the beginning rather than thorough basic research; premature translations into clinical procedures; the unbridled belief in stem cells alone ignoring the embedding environment, the need for neo-angiogenesis and the importance of biomechanics and the resulting push-back by public opinion. The latter was highlighted by the inability of the first sufficiently powered phase III clinical trial to achieve clarity with respect to bone marrow derived cells (BAMI) to recruit more than 12.5% of required patients (189). Like with other fields of cardiovascular tissue engineering, the short-cut temptation single staged cell-inoculation held may also have slowed down the clinical realization of “true” engineering approaches in myocardial regeneration. Promising developments from myocardial muscle bundles to micro-tissue spheres would have otherwise seen a much more proactive clinical translation. Given the clinical need for a successful solution, the current “death-valley” will need to be crossed with determination and in the absence of glory. While acceptance of the complexity of the pathobiology involved has been a first step toward a more scientific approach, the issue of misleading animal models also hampers success in this area of cardiovascular tissue engineering. As convenient as a murine fresh infarction model may be, the majority of patients in need of myocardial regeneration are not suffering from acute but chronic ischaemia. The “replacement fibrosis” of acute infarction (191) is distinctly different from the extracellular matrix environment building up (192) in chronic ischaemia (193) where a highly cellular interstitial environment, enriched in matricellular proteins and capable of transducing growth factor responses may be required for structural and functional recovery (193). Similar to other areas of cardiovascular tissue engineering, focused and carefully considered models like Wolfgang Schaper's chronic ischemic heart model of the 1970 (194) gave way to the more expedient acute ligation models with their exclusive focus on acute ischaemia (195, 196).

#### Two-Stage Co-culture of Scaffolds and Cells

The idea of sufficiently multiplying autologous cells *in vitro* before creating confluent functional cell layers on the blood surface of an implant again goes back to the artificial heart research of the 1960s. Adapting a method of culturing human endothelial cells that was first described in 1963 (197) it was once more Baylor College that successfully pioneered the *in-vitro* culture of autologous cells on the silastic membranes of circulatory assist devices (23) followed by the *in-vivo* proof of the antithrombogenicity of such a lining (32). At the height of pneumatically driven artificial hearts shear stress resistant confluent endothelial monolayers were created on the displacement membranes of a variety of TAHs (198–201). However, this very first tissue engineering approach utilizing cell culture never materialized as a game changer in clinical practice and quietly faded away as modern assist-devices made it obsolete.

**Figure 10:**
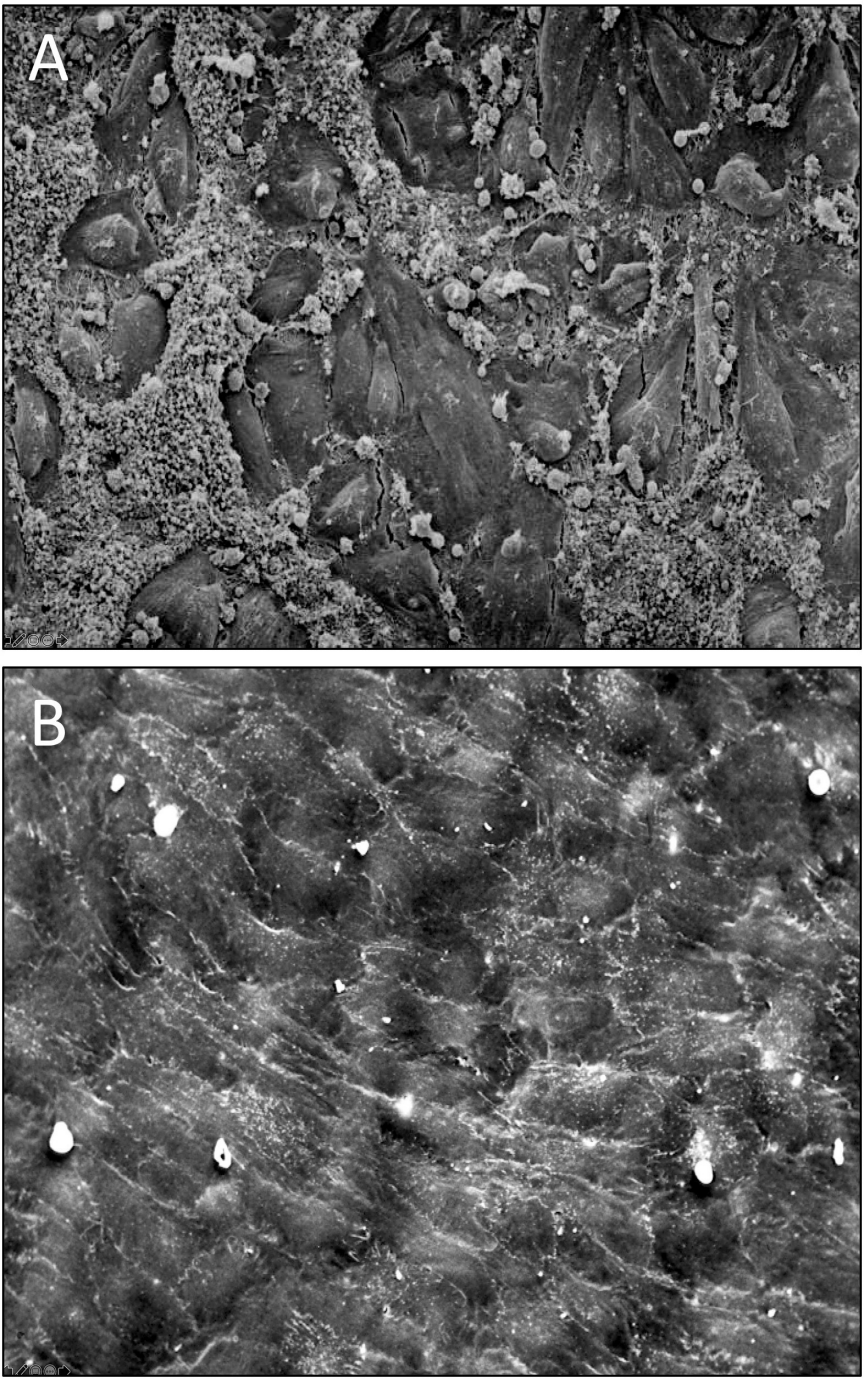
**(A)** Scanning electron micrograph of the midsegment of a 4 mm ePTFE graft, *in-vitro* enothelilised with mass-cultured, allogenic, multidonor endothelial cells after 16 days of implantation as femoral graft in a senescent Chacma Baboon. Only residual cell islands are left of an originally confluent endothelium at the time of implantation interspersed with denuded areas with densely adherent leukocytes and platelets. **(B)** Confluent monolayer of autologous endothelial cells 4 weeks after implantation of an *in vitro* endothelialised 4-mm ePTFE femoro-femoral graft into a senescent baboon. One can still recognize the underlying structure of the PTFE graft. No endothelial cell detachment was found in spite of the shear stress exposure. The antithrombogenic potential of the cultured endothelial cells was reflected by a higher patency rate and the lack of platelet or fibrin depositions. With permission from (208) and (209).

**Figure 11:**
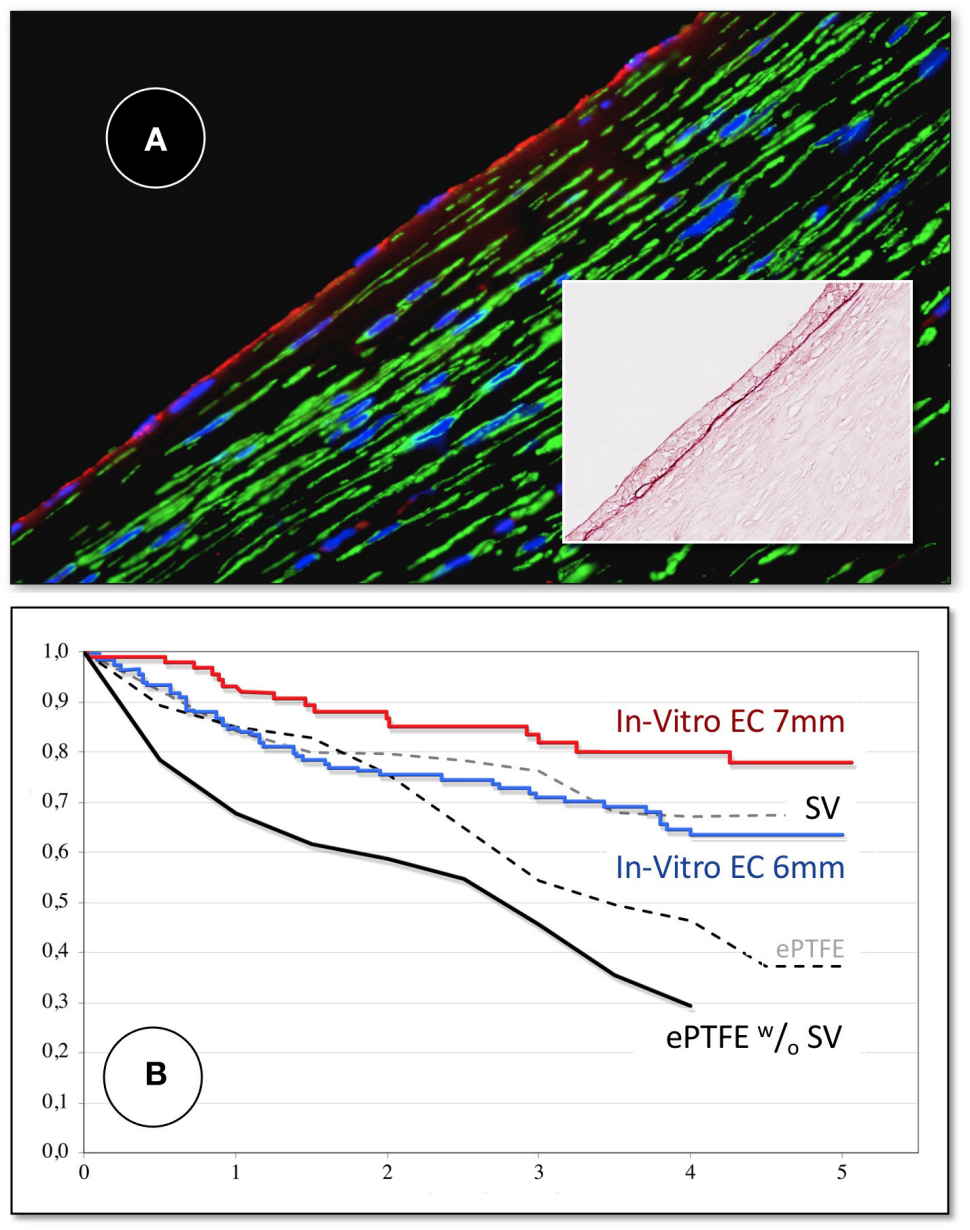
**(A)** Midgraft segment of an autologous, *in-vitro* endothelialized graft 41 months after implantation showing a confluent endothelium (CD 31) resting on layers of well-aligned actin-positive cells. A delicate intima was demarcated from the α-SMC actin positive cells by a well-defined internal elastic membrane (Insert: Orcein). **(B)** Primary patency (y-axis) over time (x-axis in years) highlighting the clinical benefit of autologous *in-vitro* endothelialisation in 6 and 7 mm femoropopliteal bypass grafts of 341 consecutive patients opposite a comparable patient group randomized to receive saphenous vein (“SV”) and ePTFE grafts (“ePTFE”) [with permission (37)]. The entire cohort of patients receiving an *in-vitro* endothelialised grafts had no saphenous vein available and as such, an ePTFE graft was an obligatory choice for each of them. Therefore, the patency of endothelialised grafts needs to be compared with that of the subgroup in the randomized Veith et al. study where an ePTFE prostheses was equally obligatory (“ePTFE ^w^/_o_ SV”). From (80) with permission.

**Figure 12:**
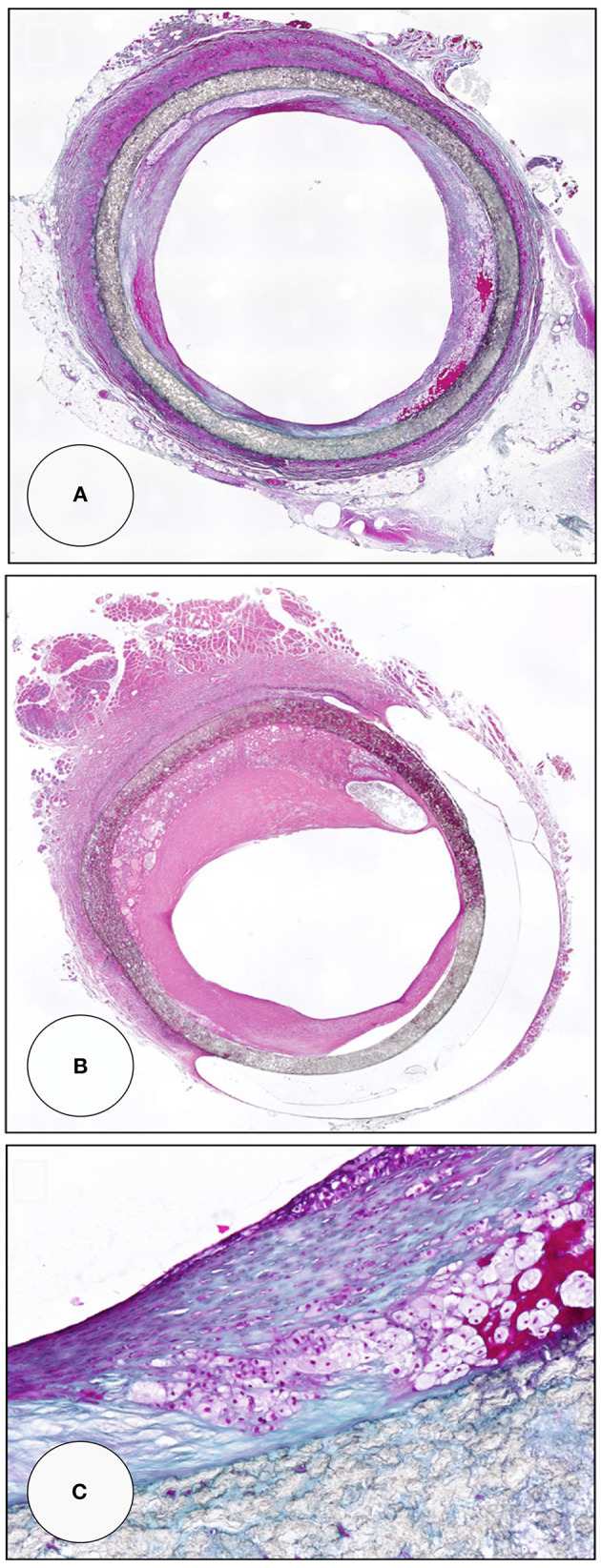
Midgraft segments of two *in-vitro* endothelialised femoro-popliteal grafts explanted at the time of re-operation for graft failure 41 months **(A,C)** and 63 months **(B)** after implantation. Both specimens contained other areas of more significant stenoses but the displayed pre-stenotic regions were packed with large islands of foam cells. Typically, the foam cells were wedged underneath pannus-like, cell-poor tissue that occasionally showed stretches of complete acellularity **(B)**. From (80) with permission.

A decade later, the group in Vienna–intimately involved in the initial clinical trials with single stage venous endothelial cell seeding of vascular prostheses (202, 203)- introduced mass-culture of autologous endothelial cells (79) to create shear-stress resistant confluent monolayers (204–206) prior to implantation. Preclinical trials confirmed the persistence and non-thrombogenicity of such tissue engineered grafts in a non-human primate model (207). An attempt to replicate this concept using cryopreserved multidonor allogenic endothelial cells in order to create “off the shelf” grafts was unsuccessful in the same senescent non-human primate model (208) (Figure 10). A clinical feasibility study was successfully done in 1989 (210) and a randomized clinical trial showed autologous *in-vitro* endothelialisation to dramatically improve the 3-year patency of femoro-popliteal and femoro-distal bypass grafts (211). Based on this evidence the group adopted this cell culture-based method as a routine procedure for all patients who had no saphenous vein available with excellent clinical outcome (212). Although a significant proportion of patients had distal reconstructions–a patient group with particularly poor prognosis–the 8 (213), 9 (214), and 12 year (215) patency rate of these grafts was continuously superior even to vein grafts. These excellent results were confirmed in more than 300 patients with a follow-up period of up to 17 years (80) (Figure 11). By then, some early limitations of this two-staged method had also been resolved. The initially experienced failure of some patients' own endothelial cells to proliferate into mass cultures had been overcome once risk factors like serum levels of lipoprotein a and triglycerides had been identified (146) and addressed (147). While delays between cell harvest and availability of the tissue-engineered grafts remained the biggest obstacle for acute clinical indications, improved mass culture techniques based on *in-situ* procedures and very-low density plating that cut out cell passaging had distinctly shortened this lag phase (79). Similarly, clinically used attachment matrices had been optimized (205, 206) culminating in an RGD-enriched, engineered lattice providing significantly enhanced shear stress resistance for the endothelium (216). The final proof of success came with the demonstration of a confluent endothelium on mid-segments of clinically explanted *in-vitro* endothelialised grafts from as early as 30 days (217) to almost 4 years after implantation (218) (Figure 11) and the histological proof of genuine arteriosclerotic changes more than 10 years after implantation (80) (Figure 12).

At the height of *in-vitro* endothelialisation of vascular grafts, the concept was also applied to bioprosthetic heart valves. Glutaraldehyde detoxification made it possible to grow endothelial cells to confluence on bioprosthetic leaflets (219–223). Such *in-vitro* lined tissue valves maintained endothelial integrity in the heterotopic primate model (224, 225) and even showed some degree of mitigation of tissue calcification (225, 226). However, when the extent of residual immunogenicity of conventional bioprostheses and its role in tissue calcification became apparent (227–229) better immune masking either through decellularization (230–233) and/or higher crosslink efficiency (234, 235) became the primary goal rather than a living endothelium. Also still in the 1980s Libby and Birinyi (236) and Pober et al. (237) had shown that under such circumstances an endothelium would augment rather than suppress an inflammatory process (236, 237) as the exposure of ECs to inflammatory cytokines, such as IL-1 or TNF-α, would cause the ECs to bind manifold more leukocytes (238) due to the induction of endothelial leukocyte adhesion molecules (ELAMs) (239). As such, an endothelium on conventional bioprosthetic leaflets even after detoxification would continually be proinflammatory (236). Attempts to reduce immunogenicity by decellularizing allografts (240–246) and xenografts (242, 247–251) before lining them with cultured endothelial cells were followed by clinical trials with *in-vitro* endothelialised, decellularized allografts (250, 252–254). When the *in-vivo* performance of equally decellularized matrices compared *in-vitro* endothelialisation with decellularization only, there was no difference (255) leading over to the later approach of non-vital decellularized native valve implants (256).

While the first two decades of tissue engineering were firmly tied to permanent scaffolds, this began to change in the 1980s. Long before anyone else, cardiovascular surgeons in Groningen had the idea of making the scaffolds gradually disappear while allowing functional tissue to develop (257–261). Pioneering this concept as early as in 1983 (261) they pre-empted key elements of the subsequent era like biodegradable polymer grafts inoculated with cultured smooth muscle cells (262). Yet, the catchy term “tissue engineering” —not coined but popularized–several years later by Joseph Vacanti, a surgeon-scientist at Boston's Children's Hospital and Robert Langer, a chemical engineer at the MIT (263) contributed to the lasting association of Boston with this approach. When they began their efforts, their underlying concept had a holistic rather than a cardiovascular claim. Indicative of the broader scope of their approach, the initial clinical problem they strove to resolve was curing diabetes through pancreatic islet transplantation (264) followed by attempts to re-grow cartilage (265). Toward, the mid 1990s, the “Boston group” had expanded this concept to heart valves (104) and shortly later also included vascular grafts (266) co-culturing embedded autologous arterial and venous cells within degradable scaffolds made of polyglactin/PGA fabric. Replacing a single valve leaflet in a sheep (104) confirmed the concept but also the previously shown superiority of autologous tissue engineered implants over their allogenic counterparts (208). However, at explantation leaflets were thicker and stiffer than native leaflets and did not have the delicate, differentiated microanatomy required for stress-reduction during opening and closing (104, 267, 268). The phenomenon of fibrosis and tissue shrinkage associated with a macrophage driven resorption process was seen in all animal models including senescent primates (269). This healing mode was well-known from the tissue reaction to resorbable surgical sutures made of the same polyglycolic- (PGA) and polylactic-acids (PLA) or polycaprolactone (PCL) materials which had been clinically used for decades (270). Attempts to minimize this cicatrition in favor of more mature valvular tissue included addressing biomechanics (271–273) as well as inadequate cell growth in the depth of the scaffolds through pulsatile bioreactors (274). Polymers were also iterated using natural polyesters like Polyhydroxyalkanoate (PHA) (271, 272) and Poly-4-Hydroxybutyrate (P4HB) (275). At the same time, the issue of the cell source also remained a challenge for two-stage culture-based approaches. Attempts to optimize the ease of harvest and the yield followed again in the footsteps of the previous era by either trying allogenic cells (276) or different autologous cell sources like bone marrow cells (275), mesenchymal stem cells (277), umbilical cord blood derived EC progenitor cells (278), prenatally harvested progenitor cells (279), human induced pluripotent stem cells (hiPSCs) (280) endothelial progenitor cells (EPC) (92) including reprogrammed and reconditioned cells (281). Different from previous eras, however, significantly more resources and internationally attracted manpower had characterized the “Boston era.” Eventually, fellows of John Mayer's group carried the program to other institutions in the early 2000s: Simon Hoerstrup to Zurich (282) [later on also collaborating with Frank Baaijens' group in Eindhoven (283)], Toshiharu Shin'oka to Tokyo (156, 157) and Christopher Breuer to Yale (later Ohio State) (160, 284). While other researchers like Laura Niklason at Duke (later Yale) (285, 286) also started their programs initially on the basis of bioreactor-based vital implants using degradable scaffolds (163), the group in Montreal went a step further. In an attempt to avoid all synthetic scaffolds and the associated problems of shrinkage and fibrosis, L'Heureux and Auger created polymer-free neo-vessels consisting of adventitial fibroblatsts, medial smooth muscle cells and an endothelium (287–291) Their extreme modern pendent are 3D printed pure stem-cell grafts (292). In clinical pilot trials with L'Heureux's AV-shunts graft dilatation was observed (288). In line with the general desire to cut out an autologous culture step and offer a product off the shelf their next generation of tissue-cultured vascular grafts was still autologous but devitalized to be storable (293) followed by a further step toward a commercial product by using devitalised allogenic cells (294). This step away from vital implants stood at the beginning of a general return to the concept of *in vivo* tissue regeneration of non-vital scaffolds originally pursued from 1968 onwards for artificial hearts (10, 11, 24, 34–36).

In retrospect, of all the vital tissue engineering concepts pursued, *in-vitro* endothelialisation was the first and for decades the last embodiment that was successfully translated into clinical practice (Figures 10–12). The successful pilot program ceased when new regulations prohibitively tightened the circumstances under which a patient's tissue may be processed as part of an implantable device outside the operating room. Although this hurdle would be surmountable by establishing integrated facilities and ISO-compatible cleanroom productions the combination of the inconvenience of not having an “off the shelf” product readily available with a protracted process that excludes acute interventions makes a revival of “vital” two-stage tissue engineering unlikely—regardless of whether the scaffold is permanent or temporary.

### Non-vital Implants

The principle behind this approach is the trust that acellular implants stimulate and direct the healing response of the body to not only become *in-situ* populated with cells but that these cells organize themselves in a way that the resulting tissue fulfills the key functions of the original structure (295). As appealing as this concept of “*in-situ*” “regeneration” or “guided tissue regeneration” is with regards to “off the shelf” products, it relies on one assumed ability: to recruit cells which can differentiate into the desired functional tissue. As practically all cardiovascular tissue engineering attempts of the past decade have coalesced toward non-vital implants, it is paramount to get clarity on their repopulation potential soon. For that, tissue sampling from the crucially important parts of the implants like the distal ventricular side of leaflets or mid-sections of long bypass grafts will be as mandatory as an independent identification and localization of ingrowing cells in order to validate this promising approach.

#### Native and *in-vitro* Grown Natural Matrices

Ironically, two diametrically opposed tissue engineering concepts of the past decades—decellularized allo and xenograft valves and pericardia on the one hand (242, 296, 297) and vital implants of cultured cells on degradable scaffolds on the other (104)—ended in congruence when the latter added a decellularization step. Now, both concepts bet on the *in-situ* re-population of allogenic or even xenogenic antigen-reduced, cell-derived matrices. A main challenge of this approach is the fine line between remnant immunogenicity (298–300) and avoidance of crosslinking to allow repopulation with host cells. The fact that a cell-free matrix can still be immunogenic has been shown (301) not least in the catastrophic failure of Synergrafts in children (302). Furthermore, interstitial cells seem to be needed to maintain the microarchitecture of the extracellular matrix (303). While the microarchitecture of the extracellular matrix of allografts is well-preserved in transplant recipients in whom the donor cells survived, the typical acellularity found in allografts from non-immune suppressed recipients is associated with a loss of microarchitecture and hyalinisation of the matrix (303).

Although the verdict is still outstanding whether such constructs will eventually get sufficiently repopulated in humans, one may in the meantime draw parallels from the experience with auto- and allograft heart valves. There, three basic facts emerged: (a) cells can remain vital in the depth of a transplanted native valve provided they are autologous and were already there at the time of implantation (304–306); (b) the survival of allogenic cells in the depth of transplanted native valves is only possible in the immune-suppressed context of heart transplantation but otherwise allograft heart valves become rapidly acellular (307); and (c) in spite of presumably representing the optimal extracellular matrix structure of a native valve, allografts remain acellular after years of implantation (303, 307). These observations indicate that in humans, even the destination matrix of a native decellularized aortic valve may remain acellular unless it already contains autologous cells at the time of implantation as is the case in autografts. Theoretically, this absence of cell ingrowth into conventional allografts could either be secondary due to post implantation matrix disturbances resulting from the death of co-transplanted cells or primarily reflect the mitigated trans-anastomotic tissue outgrowth in humans. However, even in larger animal models, the distal leaflet sections remained mostly acellular (308). Therefore, the question as to the source of cellular re-population cannot be evaded. Since all decellularized matrices present at least a temporary barrier for transmural endothelialisation, and significant fall-out healing from the circulation has remained an unfulfilled pipedream for decades (107, 108, 113, 116, 309), the partially successful “*in-situ* tissue regeneration” observed in animals may have its most likely explanation in the vigorous trans-anastomotic outgrowth potential of adjacent intimal tissue in sheep that does not exist in man. Hence, no convincing evidence going beyond facile case reports (308) has been presented yet that unambiguously demonstrated a successful autologous *in situ* re-population of decellularized native heart valves in patients. Early reports rather highlighted the unresolved issue of remnant immunogenicity of the decellularized matrix in the absence of crosslinking. CryoLife had reported partial recellularization of the distal conduit but an absence of cellularity in the leaflet after up to 11 years *in vivo* (310). This was followed by the clinical debacle with their decellularized xenografts in children (302). “Leaflet repopulation” was also reported during clinical use of the Matrix PTM line of valves, another decellularized xenograft, but the repopulating cells generally appeared to be inflammatory rather than phenotypically appropriate valve cells (302). Whether non-cardiac-derived decellularized matrices like the “CorMatrix” made from porcine small intestinal mucosa would allow to revisit the non-crosslinked xenograft concept has been decisively answered in a recent study where more than half of the valves failed (311). Together with reoperation rates in humans when used as patches (312) clinicians have been calling for a moratorium on such non-crosslinked decellularized xenografts (313, 314). Notwithstanding the conclusion that non-crosslinked decellularized xenografts are showing dangerous inflammation and failure, the excellent clinical performance of decellularized allografts over their conventional counterparts (315–317) makes it likely that the concept will prevail even if it loses the attribute of “guided tissue regeneration” upon more vigorous patho-histological analyses under strictly defined criteria.

**Figure 13:**
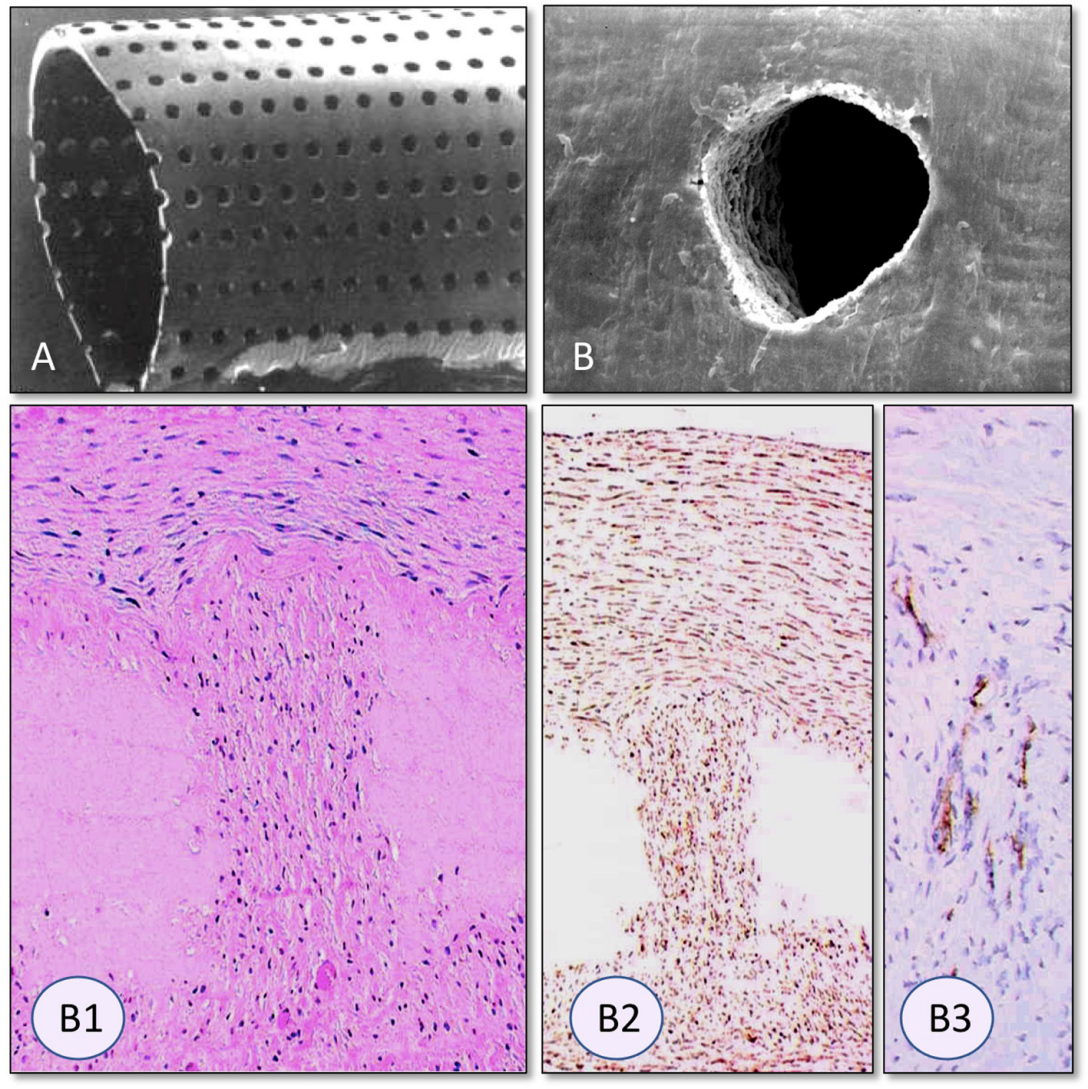
Radial Laser holes creating ingrowth permissible spaces in vascular grafts. **(A)** Laser holes in polyurethane grafts and **(B)** a decellularized arterial allograft. The clear-cut edges without tissue trauma are typical for very short-waved lasers (330). Trans-mural tissue ingrowth from the adventitia after 3 months of carotid interposition grafting in sheep. In spite of dense fully transmural tissue ingrowth through the laser perforation the decellularized allograft tissue surrounding the laser hole remained acellular (B1). Vimentin positive fibroblasts were restricted to the laser channel (B2). Within the laser hole, neovascularization was restricted to the outer half as capillary sprouting did not reach the graft lumen (von Willebrand staining) (B3). From (331, 332) and (329) with permission.

While clinical studies with decellularized natural heart valves (318–320), pericardium (296) and vascular grafts (321) have been reported for more than two decades decellularized *in-vitro* grown matrices have been tested for equally long–both as heart valves and as vascular grafts–but so far clinical trials have only been performed with vascular grafts. The concept was pioneered by Niklason's group in 2003 (322) decellularizing allogenic PGA/smooth muscle cell (SMC) constructs that were cultured in a bioreactor for 2 months. With the initial focus on dilatation resistance and patency (323) and animal implants mostly reflecting trans-anastomotic outgrowth (324) the key question as to the repopulation of the decellularized matrix in man had to wait for clinical studies. Starting in 2012 (325) sixty “Humacyte®” access grafts (325, 326) were implanted as part of a phase 2 trial and 16 tissue samples ranging from 16 to 200 weeks were histologically analyzed showing sometimes a dense repopulation of the media with α-actin positive cells (326). While showing that the decellularized matrix can be populated with mesenchymal cells, the countless wall punctures of access grafts make it difficult to ascertain whether the healing mode was transmural *per se* or required the puncture holes. A subsequent clinical femoro-popliteal bypass study eventually provided sufficiently long (28 cm) unpunctured grafts (327) to potentially clarify this issue. Of three obtained specimens, one was at the anastomosis and as such disqualified; one was at a perforation site leaving one specimen where the low-density cell infiltrates could have been evidence for a transmural repopulation. Likewise, the luminal irregularities after 24 months, reflected in angiographic diameter variations between 2.9 and 5.8 mm could alternatively be interpreted as signs of tissue formation or thrombotic appositions. Similarly, the described lack of an endothelium may have been due to the preceding attempts of balloon thrombectomies (327) or to the *a'priori* absence of an endothelium due to a lack of significant trans-anastomotic outgrowth in man and the absence of transmural endothelialisation. As such, what still remains to be proven is whether a healing mode proposed by Grabenwöger's group in Vienna as early as in 1998 (328, 329) whereby radial micro-holes greatly augmented the repopulation of decellularized matrices will eventually be required to also see surface endothelialisation on such *in-vitro* grown, decellularized matrices. It would, however, not simply be a punching-out of holes but a fine-titration between too broad ingrowth spaces that may lead to premature connective tissue maturation preventing further capillary sprouting (Figure 13) (329) and insufficient ingrowth spaces. As an alternative concept to decellularization, L'Heureux's group clinically pursued de-vitalisation through airdrying. Initially, the devitalised grafts were still partially re-vitalised by *in vitro* lining with the patient's own endothelial cells (293). This hybrid approach was followed by completely devitalised non-endothelialised allogenic grafts (“Lifeline Grafts®”) (294) eventually leading to woven textile structures using yarns of devitalised *in vitro* grown tissues (333). No repopulation, however, was ever reported in any of these grafts. Leaning on L'Heureux's earlier work (290), Syedain's group in Minneapolis using fibrin gels to create scaffold-free cell-produced decellularized vascular grafts (334) provided the most convincing repopulation evidence yet. They implanted 15 cm long AV shunts in middle-aged non-human primates (Papio Anubis) that remained unpunctured throughout. Within large animal models, Papio Anubis seems to come closest to the senescent Papio Ursinus model that was previously shown to reflect the trans-anastomotic outgrowth-stoppage (102) seen in humans (107). The study convincingly demonstrated the repopulation of the mid-graft media with mesenchymal cells that likely originated from the adventitial side (334). Yet, as it did not show traces of transmural angiogenesis and a largely absent endothelium in the mid-graft region it also highlighted once more the dependence of endothelialisation on trans-anastomotic outgrowth on these matrices—something one cannot expect in humans to occur.

Corresponding with a proven wall-repopulation but an unresolved endothelialisation mode were the suboptimal clinical results with these decellularized, bioreactor-grown vascular grafts. “Lifeline” grafts were disappointing though they were few. “Humacyte,” in contrast, had 60 access grafts implanted at 6 centers (325, 326) and 20 femoro-popliteal grafts at 3 centers (327). Primary patencies were low both in the access grafts and in the femoro-popliteal grafts. In access grafts where the 1-year primary patency is known to be between 43 and 57% for ePTFE grafts (58, 59), it was 28% for the “Humacyte” grafts (325). In above-knee femoropopliteal bypass grafts with good run-off the 2 year primary patency was 58% (327) compared to 73% in ePTFE grafts for a similar patient group in Frank Veith's multicentre study (37) and 80% for *in vitro* endothelialised 6 mm ID grafts in comparable patients (80). *In vitro* endothelialised 7 mm grafts even had a 2 year primary patency of 88% in spite of also including patients with distal reconstructions with poorer run-off (80).

During the past decade the concept of decellularized cell-produced matrices was also carried over into heart valves. The group in Zurich decellularized their well-established tissue engineered constructs of cultured human fibroblasts on a degradable scaffold (335). Using the senescent baboon model (335) and the sheep model—first for transcatheter pulmonary valve replacements (336–338) and later for orthotopic TAVIs (339)—in depth repopulation of such a matrix with mesenchymal cells was demonstrated. The patchy endothelial cover of the leaflets (158, 339), however, again hinted at the likely dependence of surface repopulation on the trans-anastomotic outgrowth seen in sheep. Furthermore, leaflet shrinkage as a consequence of repopulation emerged as an inevitable consequence of early tissue maturation (335, 336, 338) but could be addressed by shape compensation during production (340). Syedain's group in Minneapolis also tested their scaffold-free, fibrin gel-based cell-derived decellularized matrix in surgically implanted aortic valve replacements in sheep (341). Similar to others (158), repopulation with α-SMA and Vimentin positive cells was primarily seen at the leaflet base while distal leaflets remained poorly cellularized and only sporadically endothelialised.

As such, decellularized *in-vitro* grown matrices have successfully shown their ability to get repopulated by mesenchymal host cells primarily in areas of tissue contact with the host. The sub-optimal clinical performance of vascular grafts as well as the decreasing endothelial coverage with increasing distance from the nearest anastomosis, however, suggest again a dependence of the neointima formation on the trans-anastomotic outgrowth occurring in animals.

#### Synthetic (Functionalised) Scaffolds

The idea that synthetic implants contain morphogenic cues to stimulate the *in situ* regeneration of key cardiovascular structures again goes back to the early days of tissue engineering. There, the goal was to induce the *in-vivo* formation of a non-thrombogenic “neointima” on artificial hearts (10, 11, 24, 32, 34–36) and vascular grafts (342) merely on the basis of iterations of porosity and surface structures. Clinical success never materialized for the same reasons it hasn't materialized half a century later when biomechanics and molecular biology provided the tools to recapitulate refined biophysical and biochemical properties of the target tissues. (343–345). The reason for the continual absence of a clinical translation of this concept lies in decades of experimental cross-purpose design between a biologically possible tissue regeneration response and one that is actually feasible at the intended site and in the intended host environment. Although we have already highlighted that this has been the overarching dilemma of all tissue engineering approaches of the past decades, it is particularly pertinent for non-vital synthetic implants as their clinical performance depends much more on the *in-situ* formation of functional tissue (346) than any other concept. As such, an eventual break through of “*in vivo*” generation of functional tissue in synthetic scaffolds will not depend on whether the cues provided replicated nature best but whether they were based on appropriate assumptions regarding the host response.

After it had been shown that the cellular source of “fall out” healing was predominantly from endothelial precursor cells in the bone marrow (347, 348) and that these endothelial progenitor cells (EPCs) express amongst other ligands the vascular endothelial growth factor receptor VEGFR-2, as well as the CD133 and CD34 antigens (349–351) several attempts were made to augment the homing of these cells onto “non-vital” synthetic grafts by immobilizing anti-ligand antibodies on the surface (352, 353) including antibodies against chemotaxis stimulating receptors on stem cells (354). Yet, even with sporadic studies showing an enhanced recruitment of circulating cells (349, 355), without a broad consensus to focus on endothelialisation and cell population from the circulation, this approach will continue to remain an unrealised promise. As such, transmural ingrowth and endothelialisation remain the most realistic modes of tissue regeneration for synthetic scaffolds.

**Figure 14:**
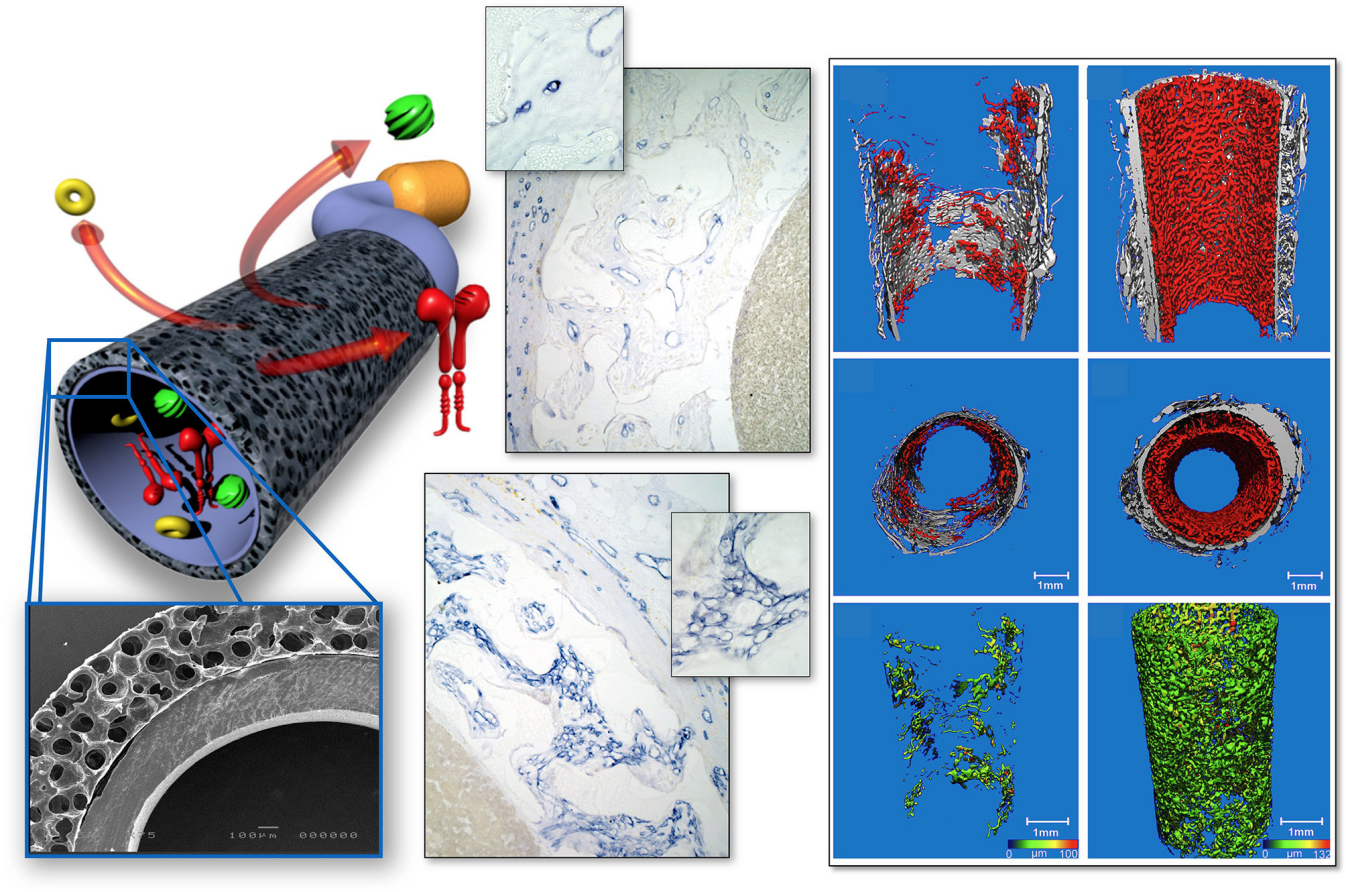
Transmural endothelialisation model in the absence of interference by trans-anastomotic endothelialisation or surface thrombus compaction. Rat subcutaneous implantation of low-porosity ePTFE-lined ingrowth permissible constructs connected to an implantable osmotic mini pump allowing well-defined administration of pro-angiogenic cues. Transmural neovascularisation is image-analytically assessed on histology and by high resolution micro-CT. From (361) with permission.

For *permanent scaffolds* to achieve successful transmural healing, ingrowth permissive spaces were shown to be a sine qua non. The minimal dimensions of such spaces were well defined at 80–400 μm^2^ (102, 356) optimally even being as large as 5,000–6,000 μm^2^ (357). Therefore, if transmural endothelialisation and healing is the goal, this requirement would exclude the use of ePTFE of <60μm internodal distance, woven Dacron and most of the electrospun polyurethane grafts unless their scaffold structures were manipulated to increase the interfibrillar spaces (357–360). Yet, making transmural endothelialisation of synthetic scaffolds a reliable occurrence also requires the suppression of the surface compaction with interstitial thrombus (107) (Figure 14). Therefore, functionalising the scaffolds with pro-angiogenic signals (361–368) must not only be seen as quantitative augmentation of angiogenesis but as the attempt to outpace the build-up of an inhibitory barrier. Encouragingly, however, thin walled ePTFE of 60–90 μm IND (102, 123, 135) whose porosity was known to provide the necessary vascular ingrowth spaces (102) was shown in non-human primates to successfully achieve transmural endothelialisation without the need for any ingrowth augmentation (102, 123, 125, 369). Regrettably, the clinical confirmation of this observation was thwarted by regulatory concerns leading to a last minute addition of a very low-porosity wrap on the outside (370) of PTFE grafts turning high-porosity into low-porosity prostheses. One of the many puzzles in tissue engineering is why the “real” clinical study never happened since, as it would have certainly provided the entire field with much needed “translational” energy.

**Figure 15:**
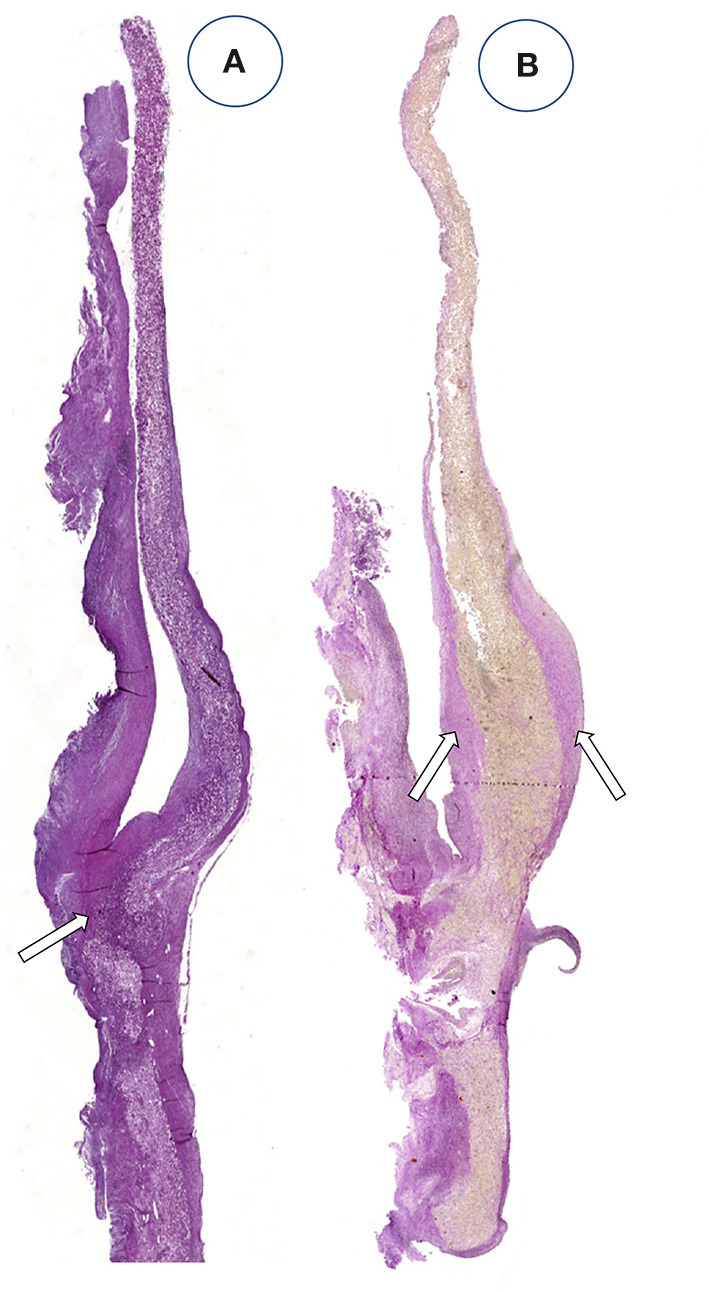
Leaflets of two different electrospun heart valves made of the biodegradable supra-molecular polymer bisurea-modified poly-carbonate (PC-BU) after 24 weeks of trans-catheter pulmonary valve replacement in the sheep. The leaflet cellularity generally decreased with distance to the leaflet base with minimal tissue deposition observed toward the free edges. Leaflet remodeling depended on stent integration in the surrounding tissue with transmural population of the leaflet base with polymer absorption and ECM deposition in the well-integrated valve **(A)** and poor cell population, surface overgrowth of tissue and a lack of collagen deposition in valve **(B)**, which was in line with its poor stent integration and migration over time indicating the dependence of the cell population of the leaflet scaffold on transmural ingrowth. From (158) with permission.

If the complexity of a clinical *in-vivo* environment makes it already difficult to accomplish transmural endothelialisation (371) in non-degradable scaffolds one needs to reckon with additional challenges in *degradable scaffolds*. There, it is less an issue of whether sufficient ingrowth spaces are pre-existent or progressively “open up” during degradation but whether the inflammatory process accompanying the break-down of the scaffold introduces additional obstacles. While “harnessing the natural inflammatory process” has been the underlying principle behind the degradation and replacement of a temporary scaffold with newly formed tissue (371–375) its effect on transmural endothelialisation has not been tested yet. Some data on electrospun heart valves confirm the presence of intramural vessels and mesenchymal cells corresponding with tissue contact (Figure 15) but a clear distinction from a trans-anastomotic origin cannot be made (158). If the latter was the case, even the intramural cell population of the scaffold would likely be different in humans. The proximity of a trans-anastomotic endothelium to a pro-inflammatory environment would trigger the active recruitment of inflammatory cells from the circulation (236, 237). The known trans-differentiation potential of such actively recruited mononuclear blood cells (85–91) and their ability to participate in the intramural cellularisation process and neo-tissue formation of a synthetic scaffold (87) makes it likely that the entire healing pattern would thus be distinctly different in humans. Ironically, a successful and rapid replacement of a biodegradable scaffold by mature mesenchymal tissue may even prematurely terminate endothelial migration (376) thereby thwarting transmural surface endothelialisation all together. While the iteration of scaffold chemistry (371, 377) and microstructure (371, 378) would allow the titration of the degradation process (371, 379) the actual clinical requirements against which to titrate remain again poorly defined in the absence of better suited animal models. Whether inserted as infra-renal interposition grafts in rats (260, 377, 380) or comparably short interposition grafts in rabbits (381, 382), dogs (383, 384), mice (385), sheep (386, 387) or pigs (388–390): trans-anastomotic outgrowth has again most likely overpowered any other form of healing in these studies on degradable scaffolds. Even if tubular structures contained valves, trans-anastomotic neointimal outgrowth provided the most probable cell source for the leaflet population observed (158, 379). In the expected absence of a neo-intima reaching the distal leaflets in humans, this may lead to situations where leaflet degradation precedes tissue formation. Clinical pilots added to this concern. Based on the equilibrium between degradation of modern supramolecular polymers originally developed in Eindhoven (379, 391) and tissue formation observed in the sheep (386) an early clinical feasibility/first in man study was commenced with pulmonary valves implanted into children. The developing post-implantation coaptation deficiency of leaflets in 11 out of 12 valves (392) hinted at a healing behavior not seen in the sheep. Without histological proof to the contrary, a lack of tissue formation in the distal leaflets will always seem the most likely explanation given the known healing deficiencies in humans. The subsequent corrective modifications of the polymer degradability (392) may have retrospectively led to well-functioning polymeric heart valves but may still defy the claim of eliciting “endogenous tissue restoration.” In a parallel clinical trial using the same material for extracardiac TCPCs (393) the close contact of the tubes with their surrounding tissue may eventually lead to successful transmural healing. However, the likelihood of obtaining sufficient representative samples within a meaningful time frame is very low in both trials: RVOT revisions increasingly happen endovascularly and TCPCs only yield samples in the rare occasion of a transplantation or death many years later. Long term animal implants published since have confirmed in similar supramolecular polymer valves that even in an animal model with excessive trans-anastomotic outgrowth like the sheep cellularisation was mainly restricted to the leaflet base while the distal leaflets remained poorly cellularised with only patchy endothelium (158).

As such, any tissue engineering approach that justifies the word “engineering” will require animal models which allow a rational and mechanistic exploration of key components of the “endogenous tissue restoration” expected at the clinical destination site. A myriad of fascinating modern scaffold materials for instance has been investigated under *in-vivo* conditions which did not emulate key cornerstones of the human situation ranging from thermoplastic polymers such as degradable polyurethanes (394); poly-e-caprolactone (PCL)–alone or in combination with poliglecaprone (395), with polydioxanone (396) or with Gelatine (397)- “supramolecular”: ureic-pyrimidone-modified polycaprolactone (158, 398) also in combination with ureic pyrimidone PEG (398) to thermoset polymers such as polyglycerol-sebacate (PGS) (399) or nanofiber scaffolds of naturally occurring polymers such as polysaccharides (cellulose) (400–402); hyaluronic acid (343); silk (385, 403), collagen (404), Elastin-like blends (405, 406), fibrin (407), or collagen-elastin (408). For none of these materials do we have an answer to two of the critical questions asked at the beginning: (1) as scaffolds, do they allow transmural endothelialisation or alternatively facilitate true fall-out endothelialisation and (2) given the absence of trans-anastomotic neointimal outgrowth in man: is scaffold degradation (eg., by blood borne inflammatory cells) balanced against neo-tissue formation to prevent a premature structure-loss in patients? The same questions need to be asked with regards to the effect of incorporated/grafted bioactive molecules such as VEGF (361, 366, 367, 409), NO (364), TGF-b (410), SFD-1 (411), and many others (368, 412) not to mention the various ingrowth gels whether they are from natural proteins (388, 407, 413, 414) or fully synthetic (415); functionalised, (416–422) and/or potentially cell selective (423). For gels, however, an important second purpose may emerge as “space-holders” further facilitating transmural endothelialisation. In this role, their presence in the interstices of the scaffolds may also prevent the build-up of impenetrable thrombus near the blood surface until the gels get replaced by ingrowing tissue.

## Conclusion

Over more than five decades, cardiovascular tissue engineering has gone through many distinctive eras from synthetic non-vital polymer implants to vital implants and back to synthetic, non-vital implants. It certainly contributed to the perception of recurring re-inventions that the distinct eras often re-established capabilities rather than integrated previously gained expertise. While scientific depth and sophistication increased over time, insight into both clinical needs and the fate of protheses in patients got lost. Amongst other developments, this growing detachment from clinical needs arose out of the transition from surgeons being the driving force to scientists. Reflecting the unprecedented progress of science in the last half-century, this transition was a natural consequence of the evolving sophistication of cardiovascular biology and material sciences. As a result, it provided an array of building blocks with a hitherto unprecedented potential of creating truly functional replacement parts for the heart and for blood vessels. Yet, empowered by this impressive armament—why are patients still not benefiting from “tissue engineered implants” unchanged from almost 60 years ago? Perhaps the *sine qua non* of product development–the user needs without which no modern device would ever pass the regulatory hurdle–had faded away. These “user needs” were originally backed by clinicians and pathologists being intimately involved with the developments. At the root of this waning association between clinical purpose, human pathobiology, and laboratory based solutions stands the diminishing feedback regarding the fate of implants in patients. During the pioneering days of cardiovascular surgery and typical for the overall spirit and often crude ethical standards of the 1960s, there was a low threshold for the clinical use of new prostheses on top of shorter life expectancies of patients. Together, they provided ample pathological evidence most prominently on the issues of trans-anastomotic outgrowth inhibition and transmural ingrowth inhibition. At that time, histological and macromorphological explant analyses of extensive clinical series often outweighed animal data. As failed grafts were surgically re-operated, clinicians had a first line opportunity to confirm the pathology. As such, no surgeon of this generation would have expected any tissue regeneration approach to rely on trans-anastomotic endothelialisation as no one would have expected a neointima that takes 10 years to cover <10 mm of a prosthesis to ever be able to endothelialise the remaining 50 cm of a distal bypass graft. Similarly, every pathologist of this era would have been able to describe the build-up of a hostile ingrowth barrier at the blood surface even in the presence of a most favorable graft porosity for trans-mural endothelialisation.

With the dawn of endovascular interventions, though, the first to lose the background knowledge of vascular pathology were the surgeons themselves. Particularly in large animal trials it were surgeons who uncritically implanted far too short interpositions to test prostheses that had clinical translation as an end goal. While the fading awareness of human pathology increasingly misled expectations regarding the tissue response at the host site something similar happened regarding the clinical needs. The detachment of scientists from a fast changing clinical practice often led to out-of-date motivations for their tissue engineering efforts. It is puzzling how exciting approaches using the most recently developed materials, cell programming or matrix engineering continue to state needs behind their research which have long outlived clinical practice.

What needs to be done? For one, adding more tools to an already overflowing, rich tool-box of modern material science, matrix- or cell biology will be unlikely to lead to a breakthrough in clinical translation. In order to achieve a break through, the key obstacle of aborted healing—be it trans-anastomotic outgrowth inhibition, the build-up of impenetrable interstitial surface thrombus, insufficient ingrowth spaces or the physical distance to cell sources–will need to be dealt with in a concerted effort. If today's clinical implants make it more difficult to identify the principles of prosthetic healing in the human cardiovascular system, sufficient historical studies are available to extract them. The principles won't have changed! Understanding the healing modes possible in patients will be a prerequisite for trying to facilitate them. Defining animal models without compromise which exclusively focus on what is possible in man will be a prerequisite for a successful iteration of the myriad of previously discovered modules and tools toward the generation of functional, mature replacement parts of the circulation. To what extent isolation models may be sufficient or more sophisticated methods may be required needs to be seen.

Overall, the re-introduction of a few forgotten principles could remove the glass ceiling that stood in the way of decades of scientific progress to move to the next level of clinical translation:

In the era of scientists and engineers having taken over the lead of most programs, modern young clinicians need to be re-integrated from the beginning to address the contemporary needs for cardiovascular tissue engineering rather than reiterate those of past decades.

Acknowledging the collective blind spot for healing modes will unfortunately be a sine qua non for succeeding.

At a time when multiple endovascular repeat interventions rather than open surgical revisions caused a paucity of experience and understanding of the patho-biology of prosthetic healing in humans a wealth of historical pathologist reports awaits to be re-discovered. The predominant materials influencing healing have hardly changed. The most sophisticated degradable leaflet materials used for tissue engineered transcatheter heart valves still largely rely on PET or PTFE for their skirts—the most likely entry path for regenerating tissue in man.

Once it is recognized what is possible and what is impossible with regards to clinical tissue regeneration it is likely that impasses will be overcome as the feasibility of the most likely healing modes in patients will guide every aspect of a program.

Once such steps have allowed to compare the numerous existing modules regarding their ability to lead to functional, fully endothelialised replacement tissue under the prevailing host conditions, the circle to the goal originally envisaged more than fifty years ago will be closed. This will still only conclude the feasibility phase of one of the great but most protracted developments in modern medicine. Yet, it will provide the impetus and focus to eventually carry this most exciting project of modern surgery to broad clinical fruition. It will be a late triumph for the generations of surgeons and scientists involved—even if in the end, the majority of these implants will be deployed by interventional means rather than conventional cardiovascular surgery.

## Author Contributions

PZ wrote the paper. MD compiled the artificial heart chapter. ND compiled the myocardial regeneration chapter. DB compiled the synthetic scaffold chapter. TP oversaw the genesis of the manuscript. All authors contributed to the article and approved the submitted version.

## Conflict of Interest

The authors declare that the research was conducted in the absence of any commercial or financial relationships that could be construed as a potential conflict of interest.
